# α‐synuclein suppresses microglial autophagy and promotes neurodegeneration in a mouse model of Parkinson’s disease

**DOI:** 10.1111/acel.13522

**Published:** 2021-11-22

**Authors:** Hai‐Yue Tu, Bao‐Shi Yuan, Xiao‐Ou Hou, Xiao‐Jun Zhang, Chong‐Shuang Pei, Ya‐Ting Ma, Ya‐Ping Yang, Yi Fan, Zheng‐Hong Qin, Chun‐Feng Liu, Li‐Fang Hu

**Affiliations:** ^1^ Department of Neurology and Clinical Research Center of Neurological Disease The Second Affiliated Hospital of Soochow University Suzhou Jiangsu China; ^2^ Jiangsu Key Laboratory of Neuropsychiatric Diseases and Institute of Neuroscience Soochow University Suzhou Jiangsu China; ^3^ Department of Pharmacology College of Pharmaceutical Sciences Soochow University Suzhou Jiangsu China; ^4^ Department of Pharmacology Nanjing Medical University Nanjing Jiangsu China

**Keywords:** autophagy, microglia, neuroinflammation, Parkinson's disease, α‐synuclein

## Abstract

The cell‐to‐cell transfer of α‐synuclein (α‐Syn) greatly contributes to Parkinson's disease (PD) pathogenesis and underlies the spread of α‐Syn pathology. During this process, extracellular α‐Syn can activate microglia and neuroinflammation, which plays an important role in PD. However, the effect of extracellular α‐Syn on microglia autophagy is poorly understood. In the present study, we reported that extracellular α‐Syn inhibited the autophagy initiation, as indicated by LC3‐II reduction and p62 protein elevation in BV2 and cultured primary microglia. The in vitro findings were verified in microglia‐enriched population isolated from *α*‐*Syn*‐overexpressing mice induced by adeno‐associated virus (AAV2/9)‐encoded *wildtype human α*‐*Syn* injection into the substantia nigra (SN). Mechanistically, α‐Syn led to microglial autophagic impairment through activating toll‐like receptor 4 (Tlr4) and its downstream p38 and Akt‐mTOR signaling because *Tlr4* knockout and inhibition of p38, Akt as well as mTOR prevented α‐Syn‐induced autophagy inhibition. Moreover, inhibition of Akt reversed the mTOR activation but failed to affect p38 phosphorylation triggered by α‐Syn. Functionally, the in vivo evidence showed that *lysozyme 2 Cre* (*Lyz2* ^cre^)‐mediated depletion of *autophagy*‐*related gene 5* (*Atg5)* in microglia aggravated the neuroinflammation and dopaminergic neuron losses in the SN and exacerbated the locomotor deficit in *α*‐*Syn*‐overexpressing mice. Taken together, the results suggest that extracellular α‐Syn, via Tlr4‐dependent p38 and Akt‐mTOR signaling cascades, disrupts microglial autophagy activity which synergistically contributes to neuroinflammation and PD development.

## Graphical abstract

Autophagy‐dependent and independent machinery synergistically contribute to hα‐Syn‐caused neuroinflammation in PD. The basal autophagy activity restricts microglia inflammation. Extracellular hα‐Syn interacts with and activates Tlr4, resulting in inflammatory responses, as well as autophagy suppression in microglia via Tlr4‐dependent p38 and Akt/mTOR signaling cascades. This impairs the inhibitory effect of autophagy on inflammation, and thus aggravating hα‐Syn‐induced inflammatory responses.

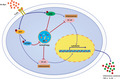

## 1 INTRODUCTION

Parkinson's disease (PD) is an age‐related neurodegenerative disorder, affecting about 2% of the population over 60. The patients with PD suffer from motor (resting tremor, rigidity, and bradykinesia) and non‐motor symptoms such as olfactory deficit, constipation, and sleep disorder. Pathologically, it is characterized by dopamine (DA) neuron losses and α‐synuclein (α‐Syn)‐abundant Lewy body or neurites formation in the substantia nigra (SN). Additionally, microglia activation, along with excessive generation of inflammatory cytokines, is reported in the brains of PD patients and animal models. Genetic and environmental factors, as well as aging, contribute to PD development. However, the cellular and molecular mechanism(s) for its pathogenesis remain to be fully elucidated.

α‐Syn misfolding and aggregation are linked to PD pathology. Under pathological conditions, this synaptic protein can be released from neurons (Jang et al., 2010; Yamada & Iwatsubo, 2018), propagating and spreading in the nervous system via cell autonomous and non‐autonomous machinery. The natural state (monomer vs. tetramer) and the structure of neuron‐released α‐Syn is controversial (Bartels et al., 2011; Nuber et al., 2018). But it is well demonstrated that extracellular α‐Syn activates microglia and inflammatory response (Jang et al., 2010; Lee et al., 2005), contributing to PD progression.

Autophagy is a highly conserved catabolic process that sequesters aberrant proteins and organelles into autophagosomes and delivers them to lysosomes for degradation and recycling. It is essential for the regulation of cellular homeostasis, stress adaptation, and survival. Several PD‐related proteins including LRRK2, PINK1, and PARKIN regulate autophagy‐lysosome pathway, and mutations of these genes result in autophagy defects (Beilina & Cookson, 2016). During the past two decades, great progress has been made in understanding the role of neuronal autophagy in PD. By contrast, the study of microglial autophagy in PD is still at infancy. Indeed, autophagy is critical for homeostatic regulation in the immune system. Dysregulation in microglial autophagy is emerging as a core regulator in brain development and diseases (Kim et al., 2017; Larabi et al., 2020; Lucin et al., 2013). Previously, we and other groups demonstrated the microglial autophagy alterations in response to lipopolysaccharide (LPS) and tumor necrosis factor‐α (TNF‐α) (He et al., 2018; Jin et al., 2018; Lee et al., 2019), and the in vitro work revealed a role of autophagy in microglia polarization and inflammation. Yet, little is known about the impact of extracellular α‐Syn on microglial autophagy. The in vivo evidence whether microglial autophagy affects neuroinflammation and DA neurodegeneration needs to be strengthened.

In this study, we reported an impairment of microglial autophagy caused by extracellular α‐Syn via toll‐like receptor 4 (Tlr4) and downstream p38 and Akt‐mTOR signaling pathways and provided the evidence that conditional knockout (cKO) of microglial *autophagy*‐*related gene 5* (*Atg5)* in mice via *lysozyme 2 Cre* (*Lyz2* ^cre^)‐mediated *Atg5* depletion, enhanced the neuroinflammation and DA neuron losses in the midbrain and exacerbated the locomotor deficits in a viral‐based α‐Syn overexpression mouse model.

## 2 RESULTS

### 2.1 Extracellular human α‐synuclein inhibited autophagy initiation in microglia

**FIGURE 1:**
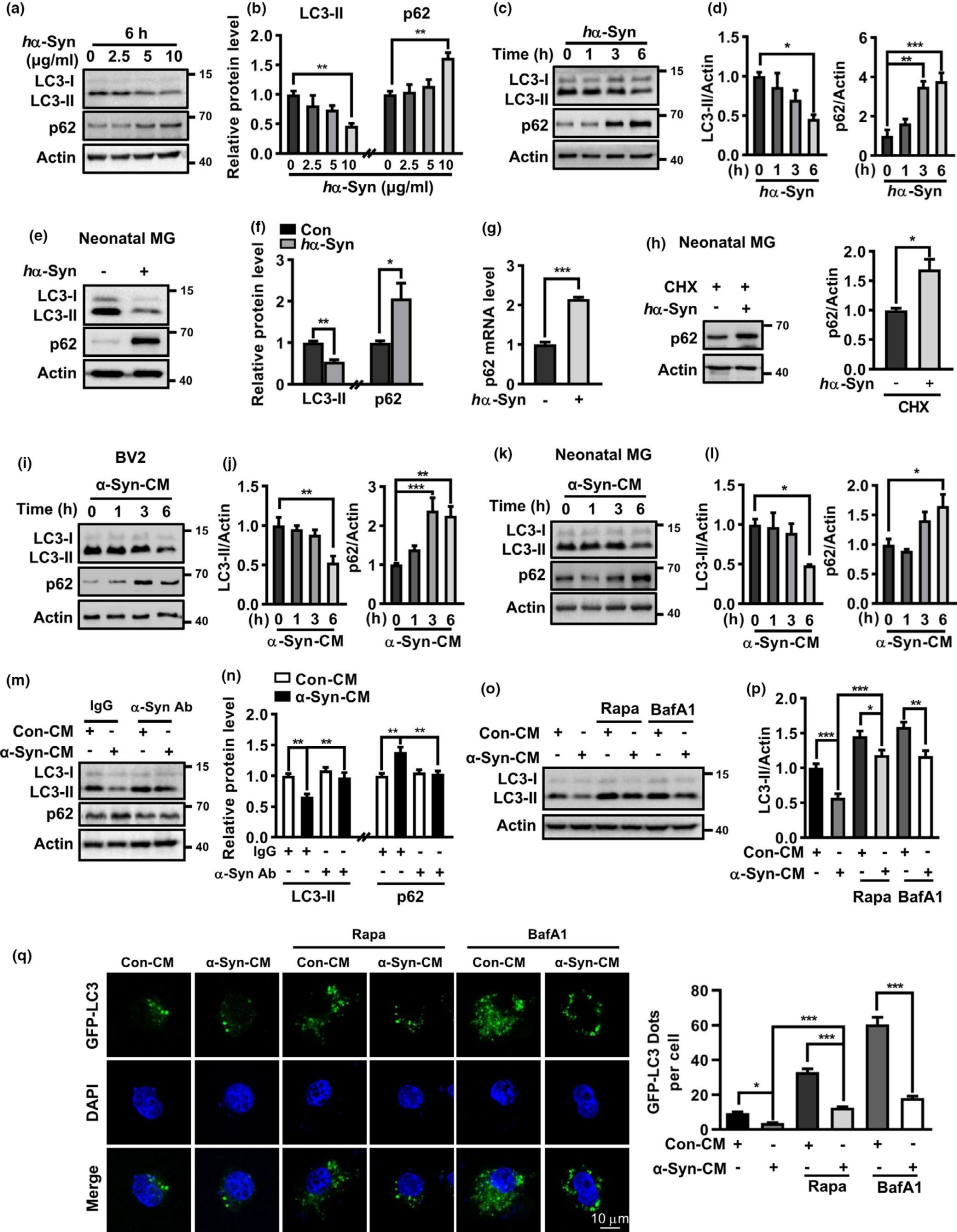
Human α‐Synuclein inhibited microglial autophagy. (a–d) Western blot and quantification of p62 protein and LC3‐II levels in *h*α‐Syn‐treated BV2 cells in dose‐ and time‐dependent manners. One‐way ANOVA with *Dunnett's post hoc*, *n* = 3–4. (e, f) p62 protein and LC3‐II levels in *h*α‐Syn (10 μg/ml, 6 h)‐treated primary microglia. Student *t* test, *n* = 3. (g) p62 mRNA level in *h*α‐Syn (10 μg/ml, 6 h)‐treated microglia. Student *t* test, *n* = 4. (h) p62 protein level in *h*α‐Syn (24 h)‐treated microglia in the presence of CHX. Student *t* test, *n* = 3. (i‐l) p62 and LC3‐II levels in *h*α‐Syn‐CM‐treated BV2 cells (i, j) and microglia (k, l). One‐way ANOVA with *Dunnett's post hoc*, *n* = 5. (m, n) Pre‐incubation with anti‐*h*α‐Syn but not IgG abolished the effect of α‐Syn‐CM on p62 protein and LC3‐II levels. Control group was treated with CM from normal PC12 cells (Con‐CM). One‐way ANOVA with *Tukey's post hoc*, *n* = 4–5. (o, p) Effect of Rapa (5 μM) or BafA1 (50 nM) on LC3‐II levels in α‐Syn‐CM‐treated BV2 cells. One‐way ANOVA with *Tukey's post hoc*, *n* = 6. (q) Imaging for GFP‐LC3 dots formation in GFP‐LC3‐transfected BV2 cells following various treatments for 6 h. Scale bar, 10 μm. At least 30 cells per group were counted. One‐way ANOVA with *Tukey's post hoc*, *p < 0.05, **p < 0.01, and ***p < 0.001

To study the effect of extracellular α‐Syn on microglia autophagy, the protein levels of two classic autophagy markers LC3‐II and p62 were examined. *h*α‐Syn appeared to dose‐dependently reduce LC3‐II but enhance p62 protein level in BV2 cells. Treatment with 10 μg/ml *h*α‐Syn reached significance (Figure 1a,b) and was applied in the following studies. A time‐dependent change of LC3‐II and p62 protein levels was observed (Figure 1c,d). Similarly, *h*α‐Syn caused LC3‐II reduction but p62 protein elevation in cultured microglia (Figure 1e,f). Moreover, *p62* mRNA level was also elevated, consistent with a recent study (Choi et al., 2020). However, *h*α‐Syn still enhanced p62 protein level even when de novo protein synthesis was blocked by cycloheximide (CHX), supporting that post‐translational increase in p62 protein level also occurred (Figure 1g,h). Next, we asked if neuron‐secreted α‐Syn had similar effects on microglia. DA cells that overexpress *h*α‐Syn were reported to release α‐Syn and stimulate microglial inflammation (Bae et al., 2012; George et al., 2019). As such, we transferred the conditioned media from *wildtype hα*‐*Syn*‐overexpressing PC12 cells (α‐Syn‐CM) to microglia culture and tested its effect on microglial autophagy. The conditioned media from normal PC12 cells (Con‐CM) served as controls. Western blotting revealed time‐dependent LC3‐II decreases and p62 protein increases in α‐Syn‐CM‐treated BV2 and microglia cells (Figure 1i–l), which were abolished when α‐Syn‐CM was pre‐incubated with *h*α‐Syn antibody but not non‐specific IgG (Figure 1m,n), implying neuron‐secreted *h*α‐Syn, in line with the recombinant protein, inhibited microglial autophagy.

Autophagy is a highly dynamic and multi‐step process. A decline in LC3‐II may result from reduced autophagosome formation or enhanced autophagic flux. To understand how *h*α‐Syn reduced LC3‐II levels, we studied LC3‐II level and LC3 dots formation changes in the absence and presence of autophagy activator rapamycin (Rapa) or lysosome inhibitor bafilomycin A1 (BafA1). As expected, Rapa alone markedly enhanced LC3‐II protein level and resulted in more GFP‐LC3 dot formation in BV2 cells transfected with GFP‐LC3. Moreover, Rapa cotreatment reversed the declines in LC3‐II and GFP‐LC3 dots formation caused by α‐Syn‐CM in microglia. BafA1 led to elevations in LC3‐II level and the number of GFP‐LC3 dots via the blockade of lysosomal degradation. Notably, α‐Syn‐CM was still able to reduce LC3‐II levels and GFP‐LC3 dots when compared to Rapa or BafA1‐alone‐treated cells (Figure 1o–q), implicating α‐Syn‐CM‐caused LC3‐II decrease mainly resulted from a decline in autophagosome formation.

### 2.2 Alterations of autophagy‐related proteins in the midbrain of *hα*‐*Syn*‐overexpressing mice

**FIGURE 2:**
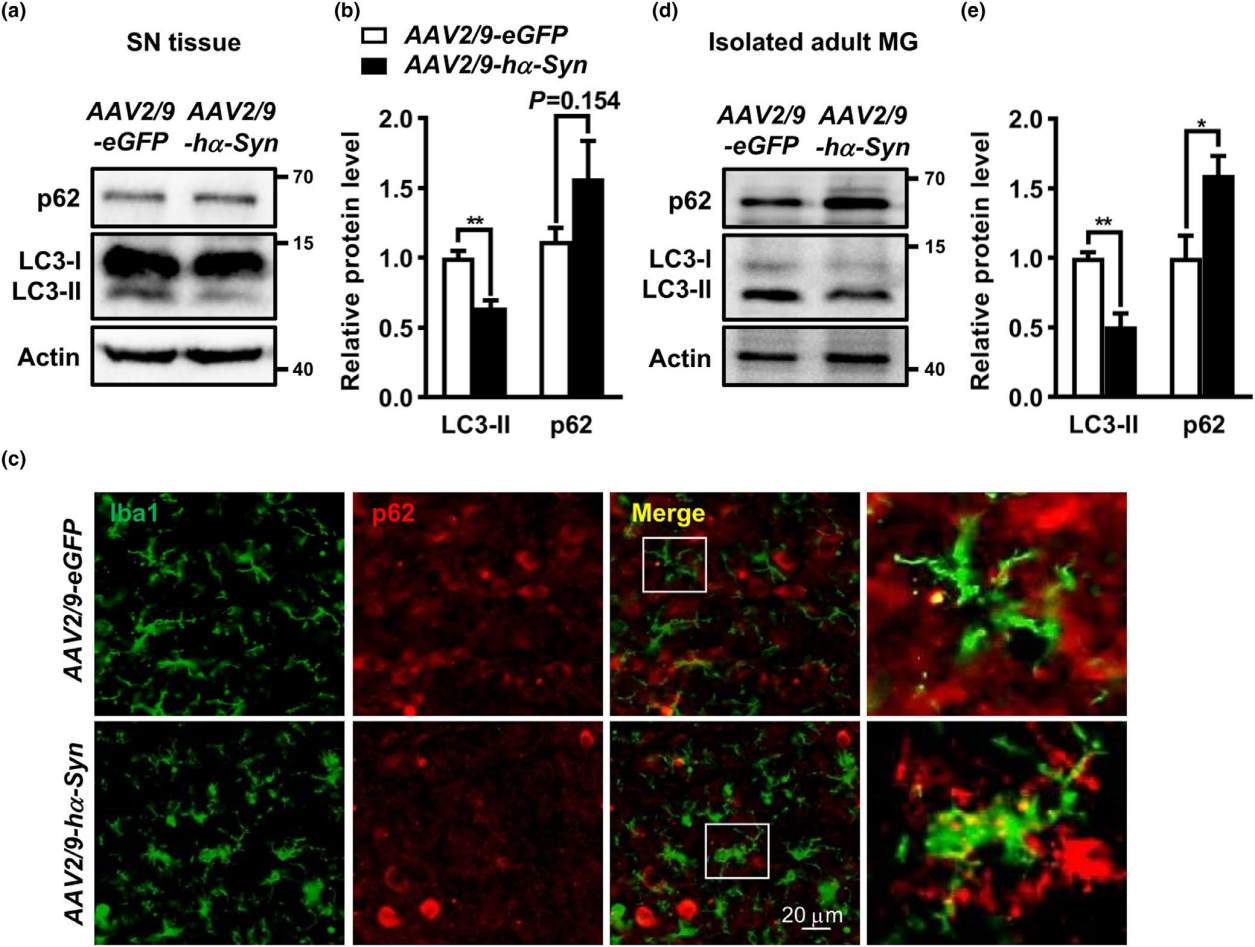
Alterations of autophagy‐related proteins in the midbrain of *hα*‐*Syn*‐overexpressing mice. (a, b) LC3‐II and p62 protein levels in the SN from C57BL/6 mice at 4 weeks following *AAV2*/*9*‐*hα*‐*Syn* or *AAV2*/*9*‐*eGFP* injection. Student *t* test, *n* = 5. (c) Midbrain sections from *AAV2*/*9*‐*hα*‐*Syn*‐ or *AAV2*/*9*‐*eGFP*‐injected mice were double stained with anti‐Iba1 and anti‐p62. White arrows indicate typical colocalization of Iba1 and p62 signal under confocal scanning. Scale bar, 20 μm. (d, e) p62 and LC3‐II protein levels in the microglia isolated from *AAV2*/*9*‐*hα*‐*Syn*‐ or *AAV2*/*9*‐*eGFP*‐injected mice at 4 weeks. Student *t* test, *n* = 3. *p < 0.05, **p < 0.01

The in vitro observations were validated in *hα*‐*Syn*‐overexpressing mice in which the SN pars compacta (SNpc) were bilaterally injected with adeno‐associated virus 2/9 (AAV2/9) carrying *wildtype hα*‐*Syn (AAV2*/*9*‐*hα*‐*Syn)* driven by *synapsin*. The mice injected with the virus carrying enhanced green fluorescent protein (eGFP) served as paralleled controls. Immunostaining displayed a predominant colocalization of eGFP or anti‐*h*α‐Syn (LB509) immunoreactivity to the cells labeled with neuron‐specific marker NeuN, but not astrocyte marker GFAP or microglia marker Iba1, implying neurons were mainly infected (Figure S1a, b). We also observed an obvious colocalization of eGFP and LB509 fluorescence to tyrosine hydroxylase (TH)‐positive neurons in the SNpc. Western blot using the antibody specifically against *h*α‐Syn (MJFR1 clone) detected *h*α‐Syn expression in the SN at 2 weeks, which gradually increased at 4 and 8 weeks after *AAV2*/*9*‐*hα*‐*Syn* injection (Figure S1c). A significant loss of DA neurons was found in the SNpc at 8 weeks (Figure S1d, e), indicating a successful establishment of a PD‐like mouse model. A lower LC3‐II level, with a mild p62 protein increase, was detected in the SN from *AAV2*/*9*‐*hα*‐*Syn*‐injected mice compared with *AAV2*/*9*‐*eGFP* group (Figure 2a, b). Double immunostaining identified a dramatic increase in p62 immunoreactive signal in Iba1^+^ microglia, which displayed an amoeboid morphology with enlarged soma, in *AAV2*/*9*‐*hα*‐*Syn*‐injected mice compared with *AAV2*/*9*‐*eGFP* group (Figure 2c), although the majority of enhanced p62 signal seemed to derive from non‐microglial cells (Figure S2). To confirm this, we acutely isolated the adult microglia and examined the autophagic markers by western blot. A remarkable increase in p62 protein, along with LC3‐II decrease, was found in the microglia population from *AAV2*/*9*‐*hα*‐*Syn*‐injected mice compared with *AAV2*/*9*‐*eGFP* controls (Figure 2d,e; Figure S3). These supported a down‐regulated autophagy activity in the microglia of *hα*‐*Syn*‐overexpressing mice, in line with in vitro data.

### 2.3 Tlr4‐dependent p38 and Akt‐mTOR activation was involved in autophagy inhibition by *h*α‐Syn

**FIGURE 3:**
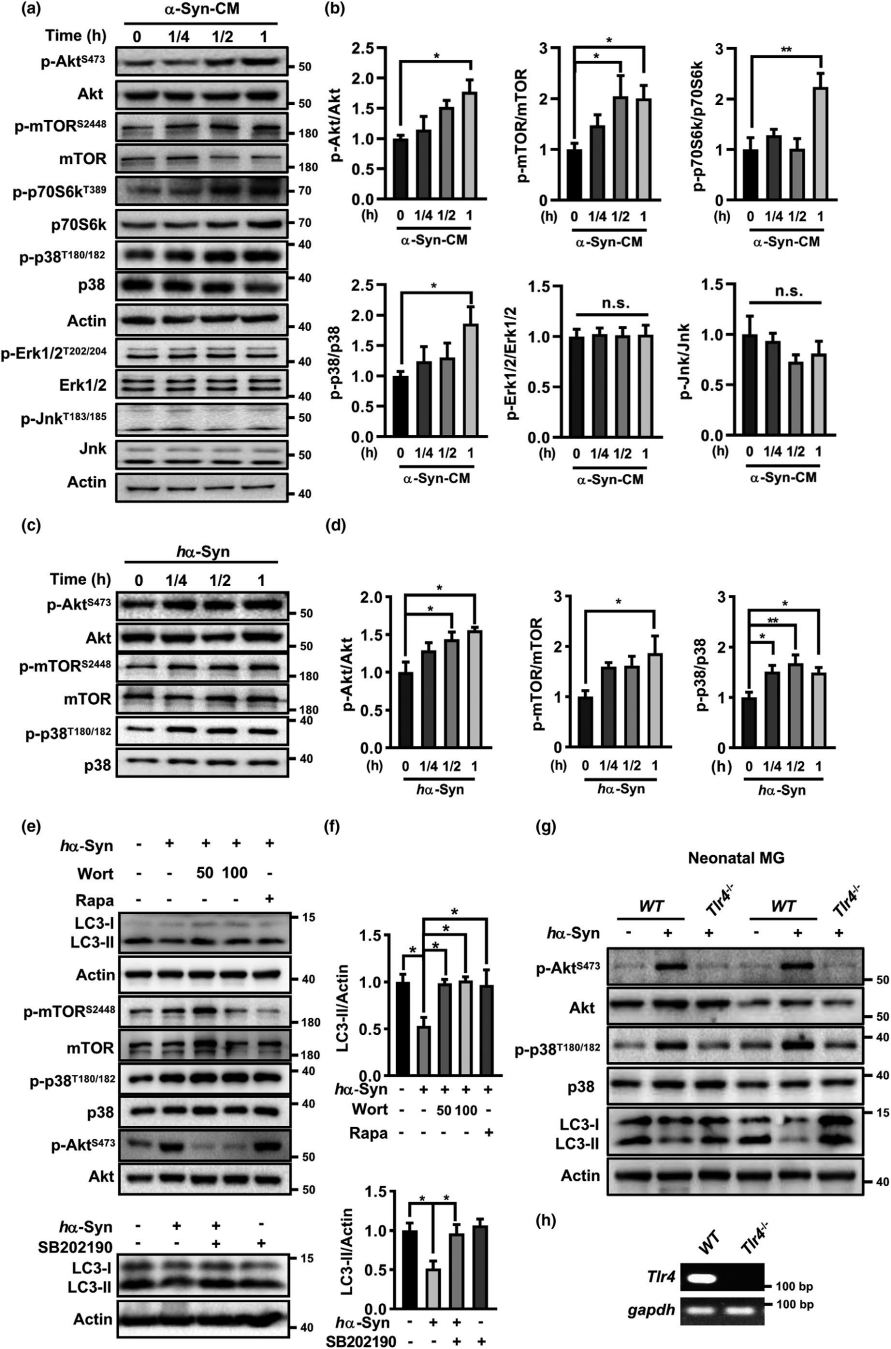
Tlr4‐dependent p38 MAPK and Akt/mTOR signaling mediated the autophagy inhibition by *h*α‐Syn. (a–d) α‐Syn‐CM‐treated BV2 cells (a, b) and *h*α‐Syn‐treated microglia (c, d) for various periods. Control group was treated with Con‐CM. Western blot analysis for various kinases phosphorylations. One‐way ANOVA with *Dunnett's post hoc*, *n* = 3–5. *p < 0.05, **p < 0.01, n.s., not significant. (e–f) *h*α‐Syn‐treated microglia (6 h) in the absence or presence of inhibitors for p38 MAPK (SB202190, 1 μM), Akt (Wort, 50, 100 nM) or mTOR (Rapa, 5 μM). One‐way ANOVA with *Tukey's post hoc*, *n* = 3–5. *p < 0.05. (g) *h*α‐Syn failed to activate p38 and Akt and inhibit autophagy in *Tlr4* ^−/−^ microglia from *Tlr4 KO* mice compared with normal microglia from wildtype (WT) C57BL/6 mice. (h) RT‐PCR verification of *Tlr4* deficiency in microglia from *Tlr4 KO* mice

We then examined the signaling for α‐Syn‐induced autophagy inhibition in microglia. First, we focused on Akt‐mTOR signaling because of its critical role in autophagy regulation. A rapid increase in Akt phosphorylation at Ser473 was found in BV2 cells following α‐Syn‐CM treatment within 1 h (Figure 3a, b). Phosphorylations of mTOR (Ser2488) and its downstream p70S6 kinase (Thr389) also enhanced. Second, we studied MAPK pathway due to its importance in α‐Syn‐induced microglial inflammation (Klegeris et al., 2008). Remarkably, α‐Syn‐CM caused a rapid increase in p38 MAPK phosphorylation early at 15 min after treatment, while Erk1/2 or Jnk phosphorylation remained unaltered. Similarly, we observed time‐dependent rises in the phosphorylation of Akt, mTOR, as well as p38 in *h*α‐Syn‐treated microglia (Figure 3c, d). Moreover, inhibition of Akt with wortmannin (Wort) prevented the mTOR activation and LC3‐II decrease caused by *h*α‐Syn. As a recent study reported that Forkhead Box O3 (FOXO3) was a downstream target of Akt for microglial autophagy inhibition by LPS (Lee et al., 2019), we examined the FOXO3 levels with western blot. However, phosphorylation of FOXO3 at Ser253 did not change up to 6 h after *h*α‐Syn treatment (Figure S4), implying a less likely involvement of FOXO3 in *h*α‐Syn‐suppressed autophagy here. Yet, mTOR inhibitor Rapa reversed *h*α‐Syn‐caused LC3‐II reduction but did not affect Akt phosphorylation, implying that mTOR was downstream to Akt activation. In addition, p38 MAPK inhibitor SB202190 reversed *h*α‐Syn‐caused LC3‐II decreases (Figure 3e, f). Inhibitors of Akt or mTOR did not affect α‐Syn‐triggered p38 MAPK activation. In sum, the results suggested that p38 MAPK and Akt‐mTOR signaling participated in *h*α‐Syn‐induced microglial autophagy inhibition.

Several studies showed that extracellular α‐Syn interacted with plasma membrane receptor Tlr4 or Tlr2 and triggered neuroinflammation in microglia (Choi et al., 2020; Fellner et al., 2013; C. Kim et al., 2013). Tlr4 may play a major role in this process because its expression is highest within human SN and upregulated in the brains of patients with PD (Drouin‐Ouellet et al., 2014; Hughes et al., 2019). To study if *h*α‐Syn‐caused microglial autophagy inhibition was mediated by Tlr4, microglia from *wildtype* and *Tlr4* ^−/−^ pups were cultured and stimulated with *h*α‐Syn. However, *h*α‐Syn did not enhance the phosphorylation of p38 or Akt, nor reduce the LC3‐II level in *Tlr4* ^−/−^ microglia compared with *wildtype* microglia (Figure 3g), indicating that *h*α‐Syn‐caused microglial autophagy inhibition was Tlr4 dependent. *Tlr4* deficiency was verified by reverse transcription PCR (Figure 3h).

### 2.4 Age‐dependent changes of microglia phenotype in microglial autophagy‐deficient mice

**FIGURE 4:**
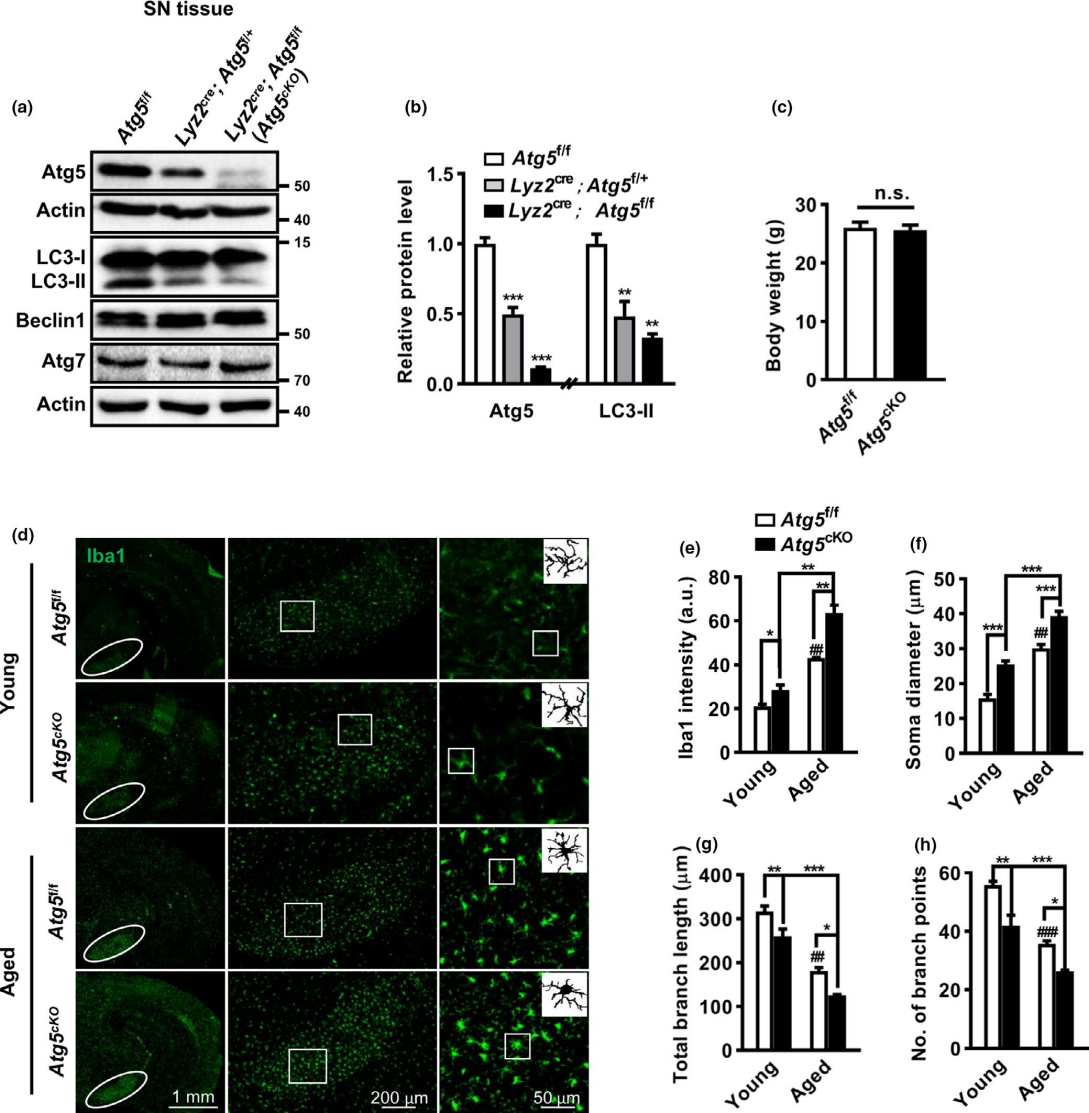
Age‐dependent changes of microglia phenotype in *Atg5* ^f/f^ and *Atg5* ^cKO^ mice. (a, b) Western blot for Atg5, LC3, Beclin1, and Atg7 in the SN of Atg5^f/f^, *Lyz2* ^Cre/+^; *Atg5* ^f/+^ and *Lyz2* ^Cre/+^; *Atg5* ^f/f^ mice. Actin served as controls. One‐way ANOVA with *Dunnett's post hoc*, *n* = 3. (c) The body weight of *Atg5* ^f/f^ (*n* = 11) and *Atg5* ^cKO^ (*n* = 9) mice at 5 months old. Student *t* test, n.s., not significant. (d) Iba1 staining and images in the SN (right panel) of young (2 months old) and aged (12–15 months old) *Atg5* ^f/f^ and *Atg5* ^cKO^ mice. (e–h) Analyses of Iba1 intensity (e, young group *n* = 6, aged group *n* = 3), soma diameter (f), branch length (g), and endpoints number (h) of microglia in the SN. a.u., arbitrary unit. *n* = 4 brains with a total of 30–50 cells per group for analysis. Two‐way ANOVA with *Tukey's post hoc*, *p < 0.05, **p < 0.01, ***p < 0.001, ^##^ *p* < 0.01, and ^###^ *p* < 0.001 versus young *Atg5* ^f/f^ mice

To understand the relevance of microglial autophagy impairment, we generated the microglial autophagy‐deficient mice by crossing *Lyz2* ^Cre^ mice with *Atg5* ^f/f^ mice followed by genotyping (Figure S5a, b). With this strategy, the autophagy gene *Atg5* was deleted in myeloid lineage‐specific lysozyme M‐expressing cells including microglia (Kim et al., 2017). Autophagy deficiency was verified by assessing Atg5, LC3‐II, Atg7, and Beclin1 protein levels in the brain homogenates. Atg5 and LC3‐II levels reduced in both heterogeneous and homogeneous *Lyz2* ^Cre^; *Atg5* ^f/f^ (*Atg5* ^cKO^) mice compared with *Atg5* ^f/f^ littermates, with more reductions in homogeneous *Atg5* ^cKO^ mice, which were then used in the following study. Atg7 and Beclin1 levels were unaffected (Figure 4a, b). Microglial autophagy deficiency was confirmed in cultured microglia from neonatal *Atg5* ^f/f^ and *Atg5* ^cKO^ mice (Figure S5c–f). Significant LC3‐II decreases and p62 protein increases were observed in microglia from *Atg5* ^cKO^ mice, in which Atg5 expression was almost undetected, indicating the successful establishment of microglial autophagy deficiency. Notably, *Atg5* ^cKO^ mice were developmentally normal and fertile, at least without any difference in body weight compared with *Atg5* ^f/f^ littermates (Figure 4c).

We then examined whether autophagy deficiency may affect microglial morphology and activation. For this purpose, brains of both young and aged *Atg5* ^f/f^ and *Atg5* ^cKO^ mice were studied by anti‐Iba1 immunostaining. Overall, a higher Iba1 intensity was found in different brain regions (SN and cortex) of *Atg5* ^cKO^ mice compared with *Atg5* ^f/f^ littermates (Figure 4d,e). Moreover, Iba1 intensities increased with ages, which was more obvious in aged *Atg5* ^cKO^ mice. A significant interaction effect was found between genotype and age (*p *< 0.05, two‐way ANOVA). We then focused on the SN, a PD‐affected region, and found the microglia in *Atg5* ^cKO^ mice, compared with those in control littermates, exhibited with enlarged soma, shortened processes, and lessened branch points. The morphological alterations also occurred with age, more obviously in aged *Atg5* ^cKO^ mice (Figure 4f–h), consistent with the Iba1 intensity results. These implicated that *Atg5* depletion may prompt microglia into a primed state and enhance their susceptibility to the second challenge, particularly with aging.

### 2.5 Autophagy modulated microglia‐mediated neuroinflammation

**FIGURE 5:**
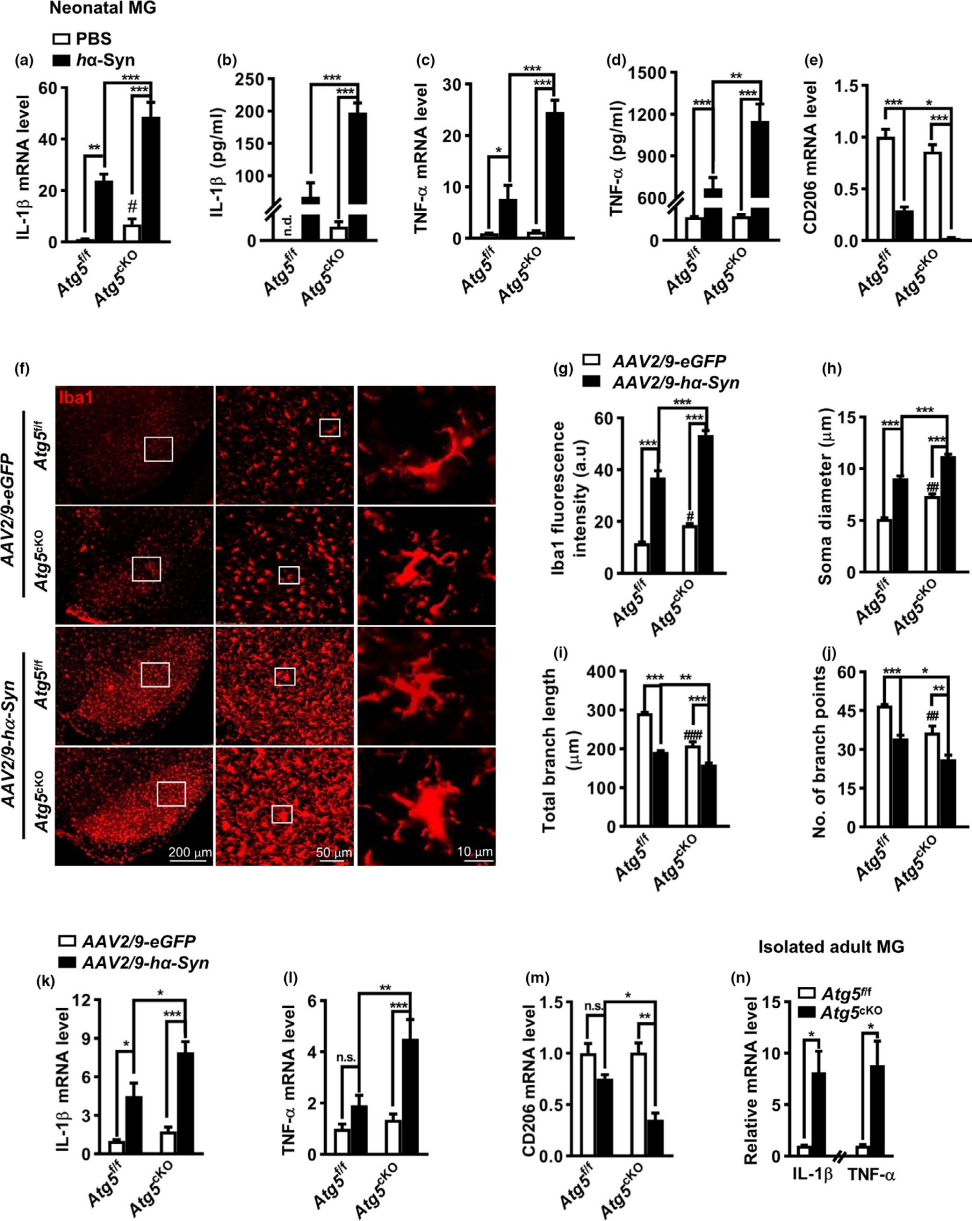
Autophagy regulated microglia‐mediated inflammation *in vitro* and *in vivo*. (a–e) Quantitative PCR of *IL*‐*1β* (a), *TNF*‐*α* (c) and *CD206* (e) mRNA levels and ELISA results (b, d) in PBS (control) or *h*α‐Syn‐treated microglia (MG) from *Atg5* ^f/f^ and *Atg5* ^cKO^ pups, *n* = 4. (f) Iba1 staining in the SN from *AAV2*/*9*‐*hα*‐*Syn*‐ or *AAV2*/*9*‐*eGFP*‐injected *Atg5* ^f/f^ and *Atg5* ^cKO^ mice at 4 weeks. (g–j) Quantification of Iba1 intensity (g, *n* = 6), soma diameter (h), branch length (i), and endpoints number (j) of microglia in (f). *n* = 4 brains with a total of 40–50 cells per group for analysis. (k–m) *IL*‐*1β*, *TNF*‐*α*, and *CD206* mRNA levels in the SN lysates of *Atg5* ^f/f^ and *Atg5* ^cKO^ mice at 4 weeks after *AAV2*/*9*‐*hα*‐*Syn* or *AAV2*/*9*‐*eGFP* injection, *n* = 3–5. Two‐way ANOVA with *Tukey's post hoc*, *p < 0.05, **p < 0.01, ***p < 0.001. ^#^ *p* < 0.05, ^##^ *p* < 0.01, and ^###^ *p* < 0.001 compared with *AAV2*/*9*‐*eGFP*‐injected *Atg5* ^f/f^ control, n.s., not significant. (n) Quantitative PCR for *TNF*‐*α* (n=3) and *IL*‐*1β* (n=4) mRNA levels in the SN from *Atg5* ^f/f^ and *Atg5* ^cKO^ mice. Student *t* test, *p < 0.05

We then explored the effect of autophagy modulation on neuroinflammation induced by *h*α‐Syn, which mimicking the pathogenesis of PD *in vitro*. The autophagy activator Rapa attenuated the increase in TNF‐α and IL‐1β mRNA levels and decrease in CD206 mRNA level induced by *h*α‐Syn‐CM in BV2 cells. The ELISA data showed that Rapa also attenuated the rises of IL‐6 and IL‐1β in the culture supernatant (Figure S6a–e). These results were verified in cultured microglia from neonatal *Atg5* ^f/f^ and *Atg5* ^cKO^ mice. Consistent with the impact of *h*α‐Syn‐CM, *h*α‐Syn resulted in an elevation of TNF‐α and IL‐1β, at both mRNA and protein levels, and a decline of anti‐inflammatory marker CD206 mRNA level in microglia from *Atg5* ^f/f^ pups, which were further exaggerated in microglia from *Atg5* ^cKO^ pups (Figure 5a–e). Remarkably, *Atg5* depletion affected the inflammation at basal conditions. A tracing amount of IL‐1β was detected in the supernatant of *Atg5* ^−/−^ microglia, while that from normal microglia was below the ELISA detection limit (<8 pg/ml). An increase in IL‐1β mRNA level was also found in *Atg5* ^−/−^ microglia although TNF‐α mRNA or protein level did not differ greatly between normal and *Atg5* ^−/−^ microglia. Generally, the *in vitro* data indicated an inhibitory effect of autophagy on microglial inflammation and implicated that an autophagy‐independent inflammatory response also existed in α‐Syn‐stimulated *Atg5* ^−/−^ microglia.

The in vivo consequence of α‐Syn‐induced microglial autophagy inhibition was investigated further. Immunostaining revealed an elevation of Iba1 intensity, with remarkable morphological changes in these Iba1^+^ cells, in the SN reticular (SNr) of both genotypes after *AAV2*/*9*‐*hα*‐*Syn* injection. Specifically, microglia displayed an amoeboid morphology, with enlarged soma and thick processes, which were different from the ramified microglia with smaller soma and numerous longer branches in control (*AAV2*/*9*‐*eGFP*) group (Figure 5f–j). These changes were more obvious in *AAV2*/*9*‐*hα*‐*Syn*‐injected *Atg5* ^cKO^ mice compared with *Atg5* ^f/f^ controls. Moreover, TNF‐α and IL‐1β mRNA levels were elevated, along with a lowered CD206 mRNA level, in the SN of *Atg5* ^cKO^ mice compared with *Atg5* ^f/f^ mice following *AAV2*/*9*‐*hα*‐*Syn* injection. A mild change in inflammation‐associated molecules (TNF‐α, IL‐1β, and CD206) mRNA level was found in the SN from *AAV2*/*9*‐*eGFP*‐injected *Atg5* ^cKO^ and *Atg5* ^f/f^ mice, probably due to the majority of non‐microglia cells in the tissue (Figure 5k–m). However, an approximate sevenfold increase in TNF‐α and IL‐1β mRNA levels was found in the microglia population acutely isolated from *Atg5* ^cKO^ mice (2 months old) relative to *Atg5* ^f/f^ controls (Figure 5n). Altogether, the data implicated *h*α‐Syn may cause inflammation in *Atg5* ^cKO^ mice, independent of autophagy inhibition. Autophagy‐dependent and autophagy‐independent machinery may synergistically contribute to *h*α‐Syn‐caused neuroinflammation in PD.

### 2.6 Microglia autophagy deficiency exacerbated DA neurodegeneration and locomotor deficits in *hα*‐*Syn*‐overexpressing mice

**FIGURE 6:**
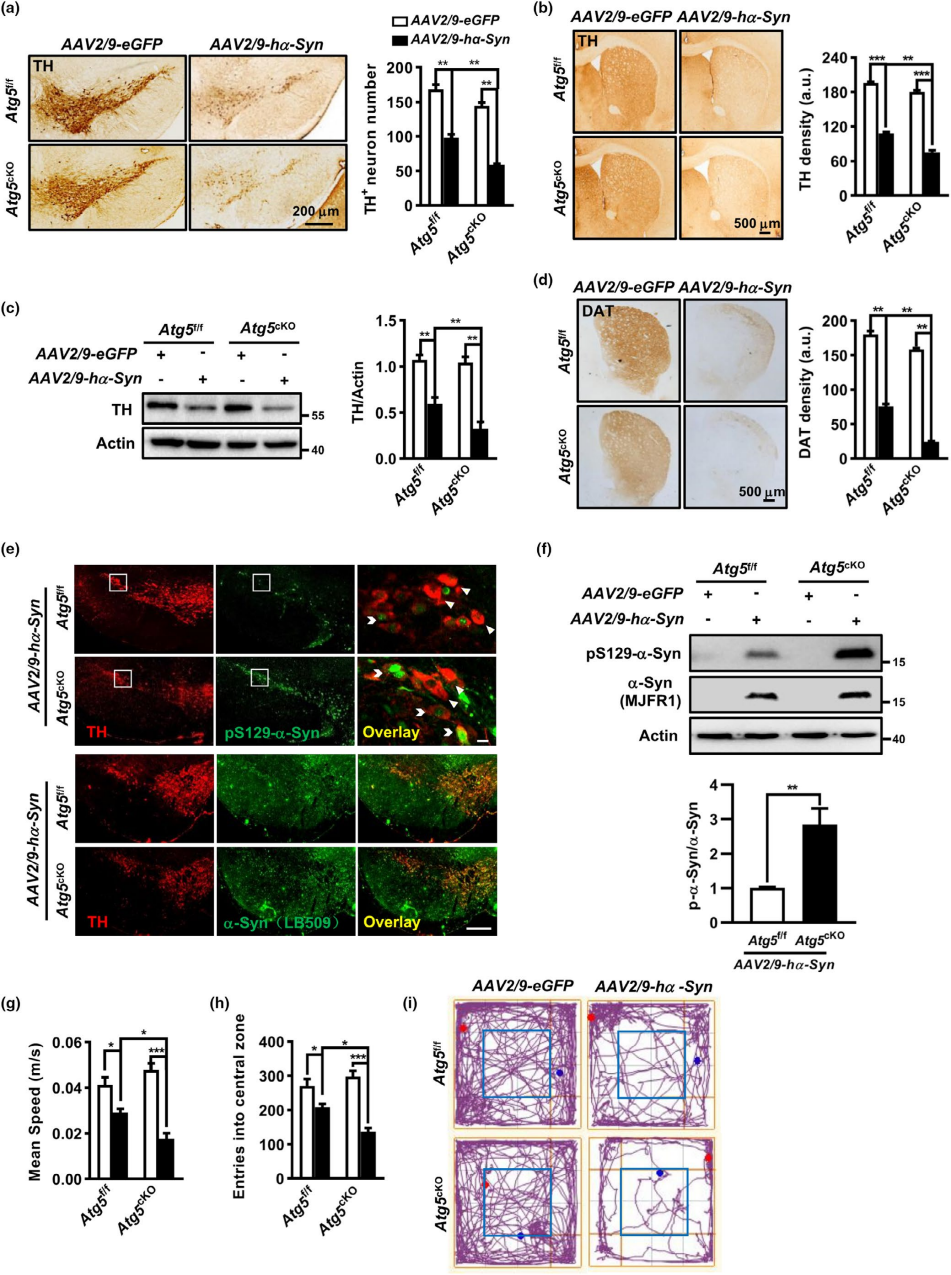
Microglia autophagy deficiency exacerbated DA neurodegeneration and locomotor deficits in *hα*‐*Syn*‐overexpressing mice. (a, b) Coronal SNpc (a) and striatum (b) sections at 8 weeks post injection and quantification for TH^+^ neuron number (*n* = 6 brains) and terminal density (*n* = 4 brains), respectively. (c) TH protein level in the SN. Two‐way ANOVA with Tukey's *post hoc*, *n* = 5. (d) Striatal sections and group data of DAT density (*n* = 4 brains). Two‐way ANOVA with Tukey's *post hoc*. (e, f) Total and phosphorylated (S129) hα‐Syn in the SN of *AAV2*/*9*‐*hα*‐*Syn*‐ *or AAV2*/*9*‐*eGFP*‐injected *Atg5* ^f/f^ and *Atg5* ^cKO^ mice at 8 weeks, assessed by immunostaining (e) and western blot (f). Scale bar, 200 μm and 10 μm. Student *t* test, *n* = 4. (g–i) Open field test for *AAV2*/*9*‐*hα*‐*Syn* (*n* = 8 for *each genotype*) *or AAV2*/*9*‐*eGFP* (*n* = 10 for each genotype) ‐injected mice. Mean speed of locomotion (g) and entries into the central zone (h) were quantified. (i) Representative trajectory pmap. Two‐way ANOVA with *Tukey's post hoc*, *p < 0.05, **p < 0.01, and ***p < 0.001

Next, we tested whether microglia *Atg5* deficiency had any effect on DA neuron losses induced by *hα*‐*Syn* overexpression. Immunostaining with anti‐TH (a DA neuron marker) showed great losses of DA neurons and terminals in the SNpc and striatum in both *Atg5* ^f/f^ and *Atg5* ^cKO^ mice at 8 weeks after *AAV2*/*9*‐*hα*‐*Syn* injection (Figure 6a, b), with more significant losses in *Atg5* ^cKO^ mice. These were verified by western blotting which showed a lower TH protein level in the SN of *Atg5* ^cKO^ mice compared with *Atg5* ^f/f^ littermates that received *AAV2*/*9*‐*hα*‐*Syn* injection (Figure 6c). We also assessed the density of DA transporter (DAT), which is located on striatal DA terminals and expressed independently of TH expression or activity. *AAV2*/*9*‐*hα*‐*Syn* injection caused a dramatic reduction of striatal DAT density, regardless of its genotype, implicating DA neurodegeneration in the nigrostriatal pathway. Compared with that in *AAV2*/*9*‐*hα*‐*Syn*‐injected *Atg5* ^f/f^ controls, the DAT density in *AAV2*/*9*‐*hα*‐*Syn*‐injected *Atg5* ^cKO^ mice was further lowered, which was almost undetectable (Figure 6d). *Atg5* ^cKO^ mice did not exhibit obvious DA neurodegeneration, only with a subtle change of TH and DAT density compared with *Atg5* ^f/f^ mice following *AAV2*/*9*‐*eGFP* injection. These data suggested that microglial *Atg5* ^cKO^ exacerbated the vulnerability of DA neurons to *hα*‐*Syn* overexpression.

α‐Syn phosphorylation at serine 129 (S129) is associated with α‐Syn pathology and reported to be elevated in PD patients (Samuel et al., 2016). Therefore, we examined the phosphorylated level of human α‐Syn (pS129‐α‐Syn). A higher pS129‐α‐Syn intensity was found in the SN of *Atg5* ^cKO^ mice compared with *Atg5* ^f/f^ littermates after *AAV2*/*9*‐*hα*‐*Syn* injection, while total α‐Syn level was comparable between two groups (Figure 6e). Double immunostaining found abundant *h*α‐Syn pathology in the remaining SNpc TH^+^ neurons after *AAV2*/*9*‐*hα*‐*Syn* injection. High magnification image showed a colocalization of pS129‐α‐Syn to TH^+^ neurons and displayed a reduced TH immunoreactivity in these co‐localized neurons (pS129‐α‐Syn^+^ TH^+^) compared with unaffected DA neurons (pS129‐α‐Syn^−^ TH^+^). The result was confirmed by western blot, revealing a higher ratio of pS129‐α‐Syn over total α‐Syn in *AAV2*/*9*‐*hα*‐*Syn*‐injected *Atg5* ^cKO^ mice relative to *Atg5* ^f/f^ controls (Figure 6f). We did not detect any band of human pS129‐α‐Syn or α‐Syn in *AAV2*/*9*‐*eGFP*‐injected mice, implying the specificity of the antibodies used.

Finally, we evaluated the animal behaviors at 8 weeks after *AAV2*/*9*‐*hα*‐*Syn* or *AAV2*/*9*‐*eGFP* injection. *Atg5* ^cKO^ mice did not show any sign of locomotor or motor coordination impairment compared with *Atg5* ^f/f^ mice even after *AAV2*/*9*‐*eGFP* injection (Figure 6g–i). *AAV2*/*9*‐*hα*‐*Syn* injection caused the reductions in locomotor speed and entries into the central area, which were more obvious in *Atg5* ^cKO^ mice than *Atg5* ^f/f^ controls. A decreased latency to fall off the rotarod was also found in *AAV2*/*9*‐*hα*‐*Syn*‐injected mice, implying the motor coordination defect. However, there was little difference in the latency to fall off the rotarod between *Atg5* ^cKO^ and their controls (Figure S7a). On the elevated plus maze, the time spent in the open arm decreased in *AAV2*/*9*‐*hα*‐*Syn*‐injected mice when compared to *AAV2*/*9*‐*eGFP*‐injected mice, with slight difference between *Atg5* ^f/f^ *and Atg5* ^cKO^ mice (Figure S7b). We also performed the tail suspension test but found no difference in the immobility time in *AAV2*/*9*‐*hα*‐*Syn*‐injected mice of both genotypes, implying the absence of depression‐like behavior (Figure S7c). Taken together, these data indicated that microglial *Atg5* depletion exacerbated the locomotor but not motor coordination impairment caused by *hα*‐*Syn* overexpression, albeit *Atg5* ^cKO^ mice did not show motor deficits up to 5 months old.

## 3 DISCUSSION

Over the last decade, our understanding on the role of neuronal autophagy in neurodegeneration deepened considerably. Yet, little is known about the contribution of microglial autophagy in PD. In this study, using BV2 cells and primary microglia treated with recombinant *h*α‐Syn and α‐Syn‐CM from *hα*‐*Syn*‐overexpressing cells, as well as adult microglia isolated from AAV2/9‐based *hα*‐*Syn*‐overexpressing mouse brains, we clearly demonstrated the microglial autophagy impairment by *h*α‐Syn, which was mediated by Tlr4‐dependent p38 and Akt‐mTOR signaling. Moreover, microglia‐specific autophagy deficiency, generated by *Lyz2* ^Cre^‐mediated *Atg5 KO*, aggravated the neuroinflammation, *h*α‐Syn aggregation, and DA neuron vulnerability in the midbrain and exacerbated the locomotor deficits in the PD mouse model induced by *hα*‐*Syn* overexpression in the SN. The findings revealed an impairment of microglial autophagy and its relevance in PD.

Extracellular α‐Syn activates microglia and neuroinflammatory responses, which contribute to PD pathogenesis. Our in vitro study showed that neuron‐released α‐Syn suppressed microglial autophagy initiation. This was verified by the in vivo data showing the accumulation of p62 protein, concomitant with LC3‐II reduction, in microglia isolated from *hα*‐*Syn*‐overexpressing mice. This provided the evidence that extracellular α‐Syn suppressed microglial autophagy activity. Additionally, we found an increased *p62* mRNA level in α‐Syn‐treated microglia. This was consistent with Choi et al. that *h*α‐Syn induced *p62* transcription, which may help to sequester the internalized α‐Syn for degradation via a process termed synucleinphagy (Choi et al., 2020). But, α‐Syn was still able to enhance p62 protein level when de novo protein synthesis was blocked, supporting that non‐transcriptional increase in p62 protein, probably due to autophagy impairment, also existed. In other words, α‐Syn can cause microglial p62 protein increases through both transcriptional upregulation and autophagy inhibition.

Seemingly, our data are inconsistent with the study by Choi et al. that 250 nM *h*α‐Syn treatment up to 9 h had little effect on total LC3‐II levels. But we consistently demonstrated that microglial autophagy impairment further promoted neurodegeneration in a synucleinopathy model. In fact, 10 μg/ml (approximately equal to 500 nM) *h*α‐Syn was applied in this study. We detected slight but insignificant LC3‐II change when microglial cells were exposed to *h*α‐Syn <10 μg/ml for shorter periods. We speculate that α‐Syn affects microglial autophagy in a concentration‐dependent manner. At lower levels, α‐Syn induces transcriptional upregulation of *p62* through NF‐κB signaling and promotes p62‐dependent α‐Syn degradation via selective autophagy; however, it also suppresses microglial autophagy and synergistically aggravates neuroinflammation at higher concentrations when α‐Syn pathology continues. Admittedly, the α‐Syn concentration applied in this study was relatively high compared with its reported level in the cerebrospinal and interstitial fluids (Stuendl et al., 2016). But, α‐Syn level may be spatiotemporally different and affected by several factors such as disease progression, neuronal activity, and circadian rhythm (Elfarrash et al., 2019; Wu et al., 2020). Our in vivo and in vitro studies consistently revealed the microglial autophagy inhibition by α‐Syn. Therefore, the α‐Syn concentration used here may still be of pathological relevance. The impact of mutant α‐Syn on microglia autophagy deserves to be studied further.

Early in 2006, two pioneer studies in nature demonstrated an essential role of basal autophagy in neuronal survival (Hara et al., 2006; Komatsu et al., 2006). Conditional deletion of *Atg7* in the SNpc DA neurons recapitulated several pathologic features of PD (Ahmed et al., 2012). The role of glial autophagy gained attention in the field of PD recently. Our study showed that conditional deletion of microglial *Atg5* by crossing *Atg5* ^f/f^ with *Lyz2* ^Cre^ lines did not induce obvious neurodegeneration or motor coordination defects in mice up to 5 months old, but promoted the neurodegeneration in different PD mouse models, consistent with previous studies (Choi et al., 2020; H. J. Kim et al., 2017; Qin et al., 2021). However, there is a discrepancy with a recent report that Itgam/CD11b‐Cre‐mediated microglial *Atg5* depletion caused early PD‐like neurodegeneration, which displayed motor coordination defects at 3 months old and cognitive decline at 8‐month‐old mice (Cheng et al., 2020). Despite this discrepancy, the microglial activation and enhanced inflammatory levels were still consistent among these studies. The discrepancy may be attributable to the expression and specificity of various Cre recombinases in the myeloid lineage, deserving to be clarified further. Given that Lyz2 is extensively expressed in myeloid cells, the impact of autophagy defects in other myeloid cells from *Lyz2* ^Cre^; *Atg5* ^f/f^ mice cannot be completely ignored. The newly generated mouse line such as *Cx3cr1* ^CreER^ which express Cre in a tamoxifen‐inducible manner may serve as a better tool to corroborate the role of microglial autophagy.

Increased IL‐1β and TNF‐α mRNA levels were identified in microglia acutely isolated from adult *Atg5* ^cKO^ mice compared with *Atg5* ^f/f^ littermates, supporting the role of Atg5‐mediated autophagy in regulating neuroinflammation. This was in line with the observations that microglia activation was more obvious in *Atg5* ^cKO^ mice compared with *Atg5* ^f/f^ under basal conditions, regardless of its age (Figure 4). Unexpectedly, we did not detect any change in TNF‐α mRNA or protein level in cultured *Atg5* ^−/−^ microglia. Probably, other compensatory mechanism that regulates TNF‐α production occurred during in vitro culture of microglia. But, the increase in IL‐1β generation was consistently found. Besides the increased transcription, we detected a small amount of IL‐1β protein in the culture supernatant of *Atg5* ^cKO^‐derived microglia, while that from *Atg5* ^f/f^ microglia was undetectable with ELISA using the same procedure. This implicated a critical role of autophagy in controlling inflammasome activation and IL‐1β generation, consistent with previous reports (Deretic & Levine, 2018; Qin et al., 2021). Heterogeneous depletion of microglia *Beclin1*, another autophagy‐related gene, also affected microglia activation and neuroinflammation in Alzheimer's disease mouse model (Houtman et al., 2019). These data support the perception that basal autophagy balances inflammation in microglia (Deretic & Levine, 2018), and autophagy defects prime microglia to be susceptible to the second challenge. Thus, α‐Syn‐suppressed microglial autophagy may synergistically contribute to the neuroinflammatory responses triggered by α‐Syn.

Autophagy is a homeostatic process with multiple effects on microglia. Besides inflammation, autophagy affects other processes including phagocytosis (Lucin et al., 2013; Nash et al., 2017), exosome secretion (Guo et al., 2020), and metabolic homeostasis (Xu et al., 2021). Microglial autophagy also plays a role in brain development. Deficient autophagy in microglia impaired synaptic pruning and caused social behavioral defects (Kim et al., 2017). The impact of autophagy on microglial phagocytosis was not explored here. However, we found more microglia activation, concomitant with morphological changes, in the brains of *Atg5* ^cKO^ mice compared with *Atg5* ^f/f^ controls under normal conditions. Whether this reflected an alteration in microglial phagocytosis needs to be investigated further.

Interestingly, we found significant changes in microglia morphology in the brains of aged mice, and these were more obvious in *Atg5* ^cKO^ compared with *Atg5* ^f/f^ mice, implicating Atg5 depletion produced an additional effect on aging‐related microglia morphology alterations. This is of great importance for age‐related neurodegenerative disorders. Microglia display diminished capacity for migration and phagocytosis and shift into a pro‐inflammatory state with ages. Although microglia‐specific changes in the expression of autophagy genes during aging still await to be clarified in future by single‐cell RNA sequencing and other novel technologies, mounting evidence suggests a critical role for autophagy in regulating age‐related changes in microglia. For example, *Atg7* depletion caused lipid droplets accumulation in microglia, which represented a dysfunctional and pro‐inflammatory state in the aging brain (Marschallinger et al., 2020; Xu et al., 2021). Moreover, defects in mitophagy, a selective autophagy for dysfunctional mitochondria, resulted in metabolic imbalance and inflammation in microglia (Fang et al., 2019; Rose et al., 2017). Therefore, α‐Syn‐caused autophagy inhibition in microglia may affect their susceptibility to aging, accelerating the progression of PD.

Autophagy and innate immunity are closely related. Autophagy modulates immune responses by regulating macrophage/microglia polarization and cytokines production. Conversely, inflammation differentially affects autophagy in macrophage and microglia, although they show a lot of similarities in origin and immune defense. For instance, the classic Tlr4 agonist LPS enhanced autophagy activity in macrophage but reduced it in microglia (He et al., 2018; Xu et al., 2007), with unknown reason for this discrepancy. β amyloid and α‐Syn, as we and others reported, disrupted the microglial autophagy processes (Cho et al., 2014; Lee et al., 2019). Different mechanisms may be involved. He et al. reported a critical role of p38 MAPK‐mediated phosphorylation of Unc‐51‐like autophagy activating kinase 1 (ULK1) in LPS‐inhibited microglial autophagy (He et al., 2018). Another study showed that Tlr4 activation by LPS suppressed microglial autophagy through PI3K‐FOXO3 pathway, independent of mTOR or MAPK1/3 (Lee et al., 2019). In this study, we found the involvement of Tlr4‐dependent p38 activation and Akt‐mTOR signaling, but not FOXO3 inhibition, in α‐Syn‐suppressed microglia autophagy. Surely, we cannot exclude the possibility that the cytokines released from α‐Syn‐activated microglia may also indirectly interfere the autophagic processes at later stages since TNF‐α and other cytokines were reported to inhibit microglial autophagy flux (Jin et al., 2018). α‐Syn was well demonstrated to interact with Tlr4 or Tlr2 in glia, which may be concentration‐ and conformation‐sensitive (Kim et al., 2013). Recent studies identified the highest expression of Tlr4 in the human SN and its upregulation in the brains of patients with PD and revealed a sensitivity of glial Tlr4 signaling to prolonged exposure to low levels of α‐Syn oligomers (Drouin‐Ouellet et al., 2014; Hughes et al., 2019). These highlighted the relevance of Tlr4 in α‐Syn‐trigged responses in microglia, in line with our data.

In sum, our findings demonstrate that α‐Syn disrupts microglial autophagy initiation via Tlr4‐dependent p38 and Akt‐mTOR signaling and reveal that microglial autophagy impairment contributes to neuroinflammation and other PD pathogenesis. Therefore, the pharmacologic and genetic strategies that aim to modulate autophagy activity in the brain may become a potential venue for PD therapy.

## 4 EXPERIMENTAL PROCEDURES

### 4.1 Chemicals and antibodies

Recombinant human α‐Syn (s7820), Rapa (V900930) and the antibodies against p62 (P0067), Atg5 (A2859), TH (T1299), and Actin (A3854) were purchased from Sigma‐Aldrich. Antibodies against phosphorylated and total MAP kinases, Akt, mTOR, and p70S6K were from Cell Signaling Technology (CST). Other primary antibodies included: LC3 (NB100‐2220, NOVUS), Iba1 (019–19741, WAKO), p‐S129 α‐Syn (ab51253, Abcam), MJFR1 clone anti‐α‐Syn (ab138501, Abcam) for western blotting and LB509 clone anti‐α‐Syn (180215, Thermo) for immunostaining, DAT (MAB369, Millipore), NeuN (MAB377, Millipore), GFAP (z033429‐2, DAKO), phosphorylated FOXO3 (AP0684) and FOXO3 (A0102, ABclonal). The inhibitor for p38 (SB202190) and PI3K (Wort) was purchased from Selleck, BafA1 from Abcam, and CHX from Med Chem Express.

### 4.2 Cell culture

Primary microglia were prepared from P1‐P3 pups as we previously reported (Yuan et al., 2018). Briefly, cortex was isolated and dissociated in 0.05% trypsin followed by filtering through a 70‐μm strainer. After centrifugation, the pellet was resuspended and cultured in Dulbecco's modified Eagle's medium (DMEM) containing 1% penicillin/streptomycin, GlutaMax^TM^, and 10% fetal bovine serum (FBS). After culture for 1–2 weeks, microglia were detached off the flasks by orbital shaking at 180 rpm for 2 h and seeded into dishes for experimentation. BV2 cells were maintained in DMEM supplemented with 10% FBS and 1% penicillin/streptomycin mixture in a 37°C incubator. The reagents for cell culture were obtained from Gibco.

### 4.3 Preparation of α‐Syn‐enriched conditioned media

To prepare α‐Syn‐enriched conditioned media (α‐Syn‐CM), PC12 cells were infected with *pLentiVENUS*‐*YFP*‐*hα*‐*Syn* as previously reported (Wang et al., 2015). At 24 h post infection, media were replaced with fresh DMEM and incubated for 48 h. The cell‐free culture media were harvested and centrifuged at 10,000 g using 10 kDa cut‐off filters (Millipore). The resulting supernatants were referred to as α‐Syn‐CM for microglia treatment further. To verify if CM‐contained α‐Syn affected microglial autophagy, CM was pre‐incubated with 2 μg/ml anti‐α‐Syn antibody (#2642, CST) or non‐specific IgG for 10 min before being transferred to microglia culture.

### 4.4 Western blotting

Cells were lysed in RIPA buffer (25 mM Tris, 150 mM NaCl, 5 mM EDTA, 1% NP‐40, pH 7.5) with a protease/phosphatase inhibitor tablet. Brain tissues were homogenized in the lysis buffer at 1:10 (w/v) and centrifuged at 13,000 rpm for 30 min. Western blot analysis was performed as described previously (Yuan et al., 2018). For α‐Syn phosphorylation analysis, the membranes were fixed in 4% paraformaldehyde (PFA) before subjected to blocking and incubation with anti‐p‐S129 α‐Syn.

### 4.5 GFP‐LC3 plasmid transfection and punctate visualization

To monitor microglial autophagy activity, BV2 cells were transiently transfected with eGFP‐LC3 plasmid (Addgene) using lipofectamine 2000. At 48 h post transfection, cells were subjected to indicated treatments for 6 h. Cells were fixed in 4% PFA for 10 min, stained with DAPI, and observed under a confocal microscope (LSM700; Carl Zeiss).

### 4.6 Animals and stereotaxic surgery

To induce α‐Syn overexpression *in vivo*, the SN of 8‐week‐old male C57BL/6 mice (SLRC Laboratory) were bilaterally injected with *AAV2*/*9*‐*hα*‐*Syn* (1.28E+10 v.g) driven by the promoter synapsin (Obio). For control group, mice were injected with *AAV2*/*9*‐*eGFP*. For surgery, mice were anaesthetized with 2% isoflurane and injected with 1.5 μl of *AAV2*/*9*‐*hα*‐*Syn* or *AAV2*/*9*‐*eGFP* at the rate of 0.3 μl/min, with the coordinates at AP −3.0 mm, ML ±1.25 mm, and DV +4.5 mm relative to the bregma (Figure S1a). The needle was left in place for 5 min before being retracted.

To generate mice lacking *Atg5* in microglia, we crossed *Lyz2* ^Cre^ mice (Model Animal Research Center, MARC, Nanjing, China) with *Atg5* ^f/f^ mice (kindly provided by Dr. Noboru Mizushima, Tokyo Metropolitan Institute of Medical Science, and obtained from RIKEN BioResource Center, Japan, #02975). Littermates were used in all experiments. Genotyping was conducted as previously reported (Hara et al., 2006). The breeding strategy and genotyping results are shown in Figure S2. *Tlr4* KO mice were obtained from Nanjing University MARC. Animals were bred at the Laboratory Animal Facility in Soochow University under specific pathogen‐free conditions and maintained at a constant ambient temperature, with access to tap water and food ad libitum. All animal experiments were carried out in accordance with the guidelines of the Institutional Animal Care and Use Committee of Soochow University.

### 4.7 Adult microglia isolation

Adult microglia were isolated according to a reported protocol (Galatro et al., 2017) with minor modifications. Briefly, whole brains were isolated from mice after intracardial perfusion with chilled PBS and homogenized in 5 ml ice‐cold serum‐free DMEM. Homogenates were filtered through a 70‐µm cell strainer and centrifuged with 1000 ×g for 10 min at 4°C, followed by suspension with 22% Percoll and centrifuge again to remove the myelin fragments. After that, the pellet was resuspended in 4 ml 37% Percoll and slowly covered on the top of 4 ml 70% Percoll and centrifuged with 1000 x g for 30 min with no brake. The 70–37% interphase was carefully collected and resuspended with 3 times volume of Hank's balanced salt solution (HBSS) followed by centrifuge at 800 ×g for 10 min at 4°C. The pellet was collected as microglia‐enriched population, and the purity was determined via flow cytometry (Figure S3).

### 4.8 Quantitative polymerase chain reaction (qPCR)

Total RNAs were reverse transcribed using the cDNA synthesis kit (Thermo Fisher Scientific). Target genes were amplified using SYBR Green PCR Master Mix (Q711, Vazyme) with specific primers in Table 1. Amplification of DNA products was performed using the 7500 Real‐Time PCR system (Applied Biosystems). Relative mRNA levels were calculated according to the 2**^−△△Ct^** method. All delta Ct values were normalized to GAPDH.

**TABLE 1:** Primers of the genes tested

Gene	Primers	Sequence
*cd206*	Forward primer	5′‐TCTTTGCCTTTCCCAGTCTCC‐3′
	Reverse primer	5′‐TGACACCCAGCGGAATTTC‐3′
*gapdh*	Forward primer	5′‐GAAGGTCGGTGTGAACGGAT‐3′
	Reverse primer	5′‐AATCTCCACTTTGCCACTGC‐3′
*il‐1β*	Forward primer	5′‐ TGGAAAAGCGGTTTGTCTTC‐3′
	Reverse primer	5′‐TACCAGTTGGGGAACTCTGC‐3′
*p62*	Forward primer	5′‐GAAGCTGCCCTATACCCACA‐3′
	Reverse primer	5′‐CCCGATGTCGTAATTCTTGGTC‐3′
*tlr4*	Forward primer	5′‐ACCTGGCTGGTTTACACGTC‐3′
	Reverse primer	5′‐CTGCCAGAGACATTGCAGAA‐3′
*tnf‐α*	Forward primer	5′‐CTGAGGTCAATCTGCCCAAGTAC‐3′
	Reverse primer	5′‐CTTCACAGAGCAATGACTCCAAAG‐3′

### 4.9 Cytokine measurement

The contents of TNF‐α and IL‐1β were determined by the enzyme‐linked immunosorbent assay (ELISA) kits from eBioscience, while IL‐6 were quantified using the kit from R&D system according to the manufacturer's instructions.

### 4.10 Immunostaining

Mice were intracardially perfused with 4% PFA and post‐fixed overnight. Following dehydration in a serial of 10%–30% sucrose solution, 18‐μm thick sections were coronally cut using a cryostat (Leica). For 3,3‐diaminobenzidine (DAB) staining, free‐floating sections were pretreated with 3% hydrogen peroxide for 10 min before blocking in 5% BSA with 0.25% Triton X‐100 and incubated with anti‐TH (1:1000) or anti‐DAT (1:500) at 4°C overnight. Next, sections were incubated with biotinylated secondary antibody and streptavidin‐horseradish peroxidase complex sequentially. TH and DAT immunoreactivities were visualized using DAB kit (GK500705, Gene Tech). For fluorescent staining, slices were blocked in 0.1% Triton X‐100 with 10% BSA for 1 h and incubated with anti‐Iba1 (1:1000), anti‐GFAP (1:1000), p62 (1:500), and anti‐p‐S129 α‐Syn (1:500) overnight at 4°C. After rinsing three times in PBS, slices were incubated with secondary antibody (Thermo Fisher Scientific) in dark for 2 h, briefly rinsed, and mounted with the media containing DAPI (H‐1200, Vector Laboratory). Images were taken under a bright‐field microscope or confocal microscope (Carl Zeiss).

The number of TH^+^ neurons was counted using the “cell counter” plug‐in in Fiji software and corrected by a researcher blinded to testing groups. Every fifth section throughout the SN was counted, and at least four sections per brain were included for quantification. For striatal TH or DAT intensity, at least four sections per brain were included for analysis using the ImageJ software. The microglial morphological analyses were performed using Fiji plug‐in in AnalyzeSkeleton (2D/3D) software according to a previously published protocol (Young & Morrison, 2018).

### 4.11 Behavior tests

The mice were subjected to several behavior tests at 8 weeks after stereotaxic injection, by the researcher blinded to treatment groups. All tests were performed between 9:00–17:00. The open field consisted of a rectangular plastic box (40 cm × 40 cm × 40 cm) and was subdivided into peripheral and central area, where the central zone included a square of 20 cm × 20 cm. For each session, the mouse was placed into the edge of an open field and allowed to freely explore for 10 min under dim light. The field was thoroughly cleaned with ethanol between each session. A video tracking system (ANY‐Maze software) recorded the locomotor activity. The procedures for other tests were provided in the supple information.

### 4.12 Statistical analysis

Data are presented as mean ± SEM of at least three independent experiments unless indicated. The statistical analysis between two groups was determined using Student unpaired *t* test. For multiple‐group comparisons, statistical significance was determined by one‐way analysis of variance (ANOVA) followed by *Tukey*, *Dunnett post hoc test*, or two‐way ANOVA followed by Tukey post hoc analysis as described in the legend using GraphPad Prism 8 software. Differences with *p* value < 0.05 were considered statistically significant.

## CONFLICTS OF INTEREST

We have no potential conflict of interest to be disclosed.

## AUTHOR CONTRIBUTIONS

LFH, HYT, BSY, and XOH conceived the study and designed the experiments. HYT, BSY, and XOH involved in acquisition of data. XJZ involved in adult microglia isolation. CSP and YTM involved in primary microglia culture. ZHQ, YF, and CFL contributed to the revision of the manuscript for important intellectual content. LFH, HYT, BSY, and XOH assembled the figures, and LFH wrote the manuscript with input from all authors. LFH and YPY involved in obtained funding.

## Supporting information

Fig S1

Fig S2

Fig S3

Fig S4

Fig S5

Fig S6

Fig S7

Supplementary Material

## Data Availability

The data that support the findings of this study are available from the corresponding author upon reasonable request.
