# Supplementary figures and images for: α‐synuclein suppresses microglial autophagy and promotes neurodegeneration in a mouse model of Parkinson’s disease
Source: Aging Cell. 2021 Nov 22;20(12):e13522. doi: 10.1111/acel.13522 (PMC8672776; doi:10.1111/acel.13522)

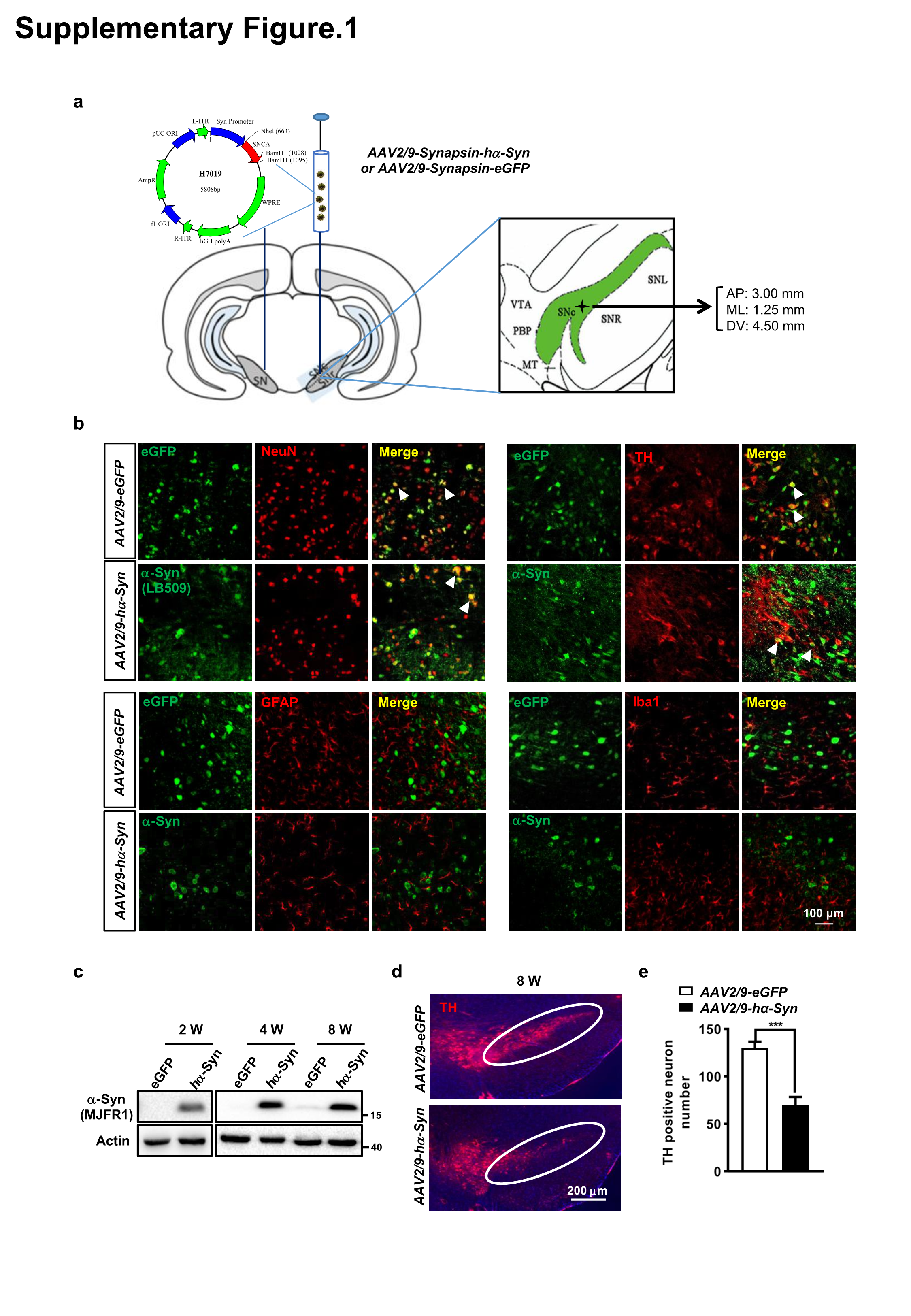

Supplement: Supplementary file 1 — Fig S1 [file ACEL-20-e13522-s007.Tiff]

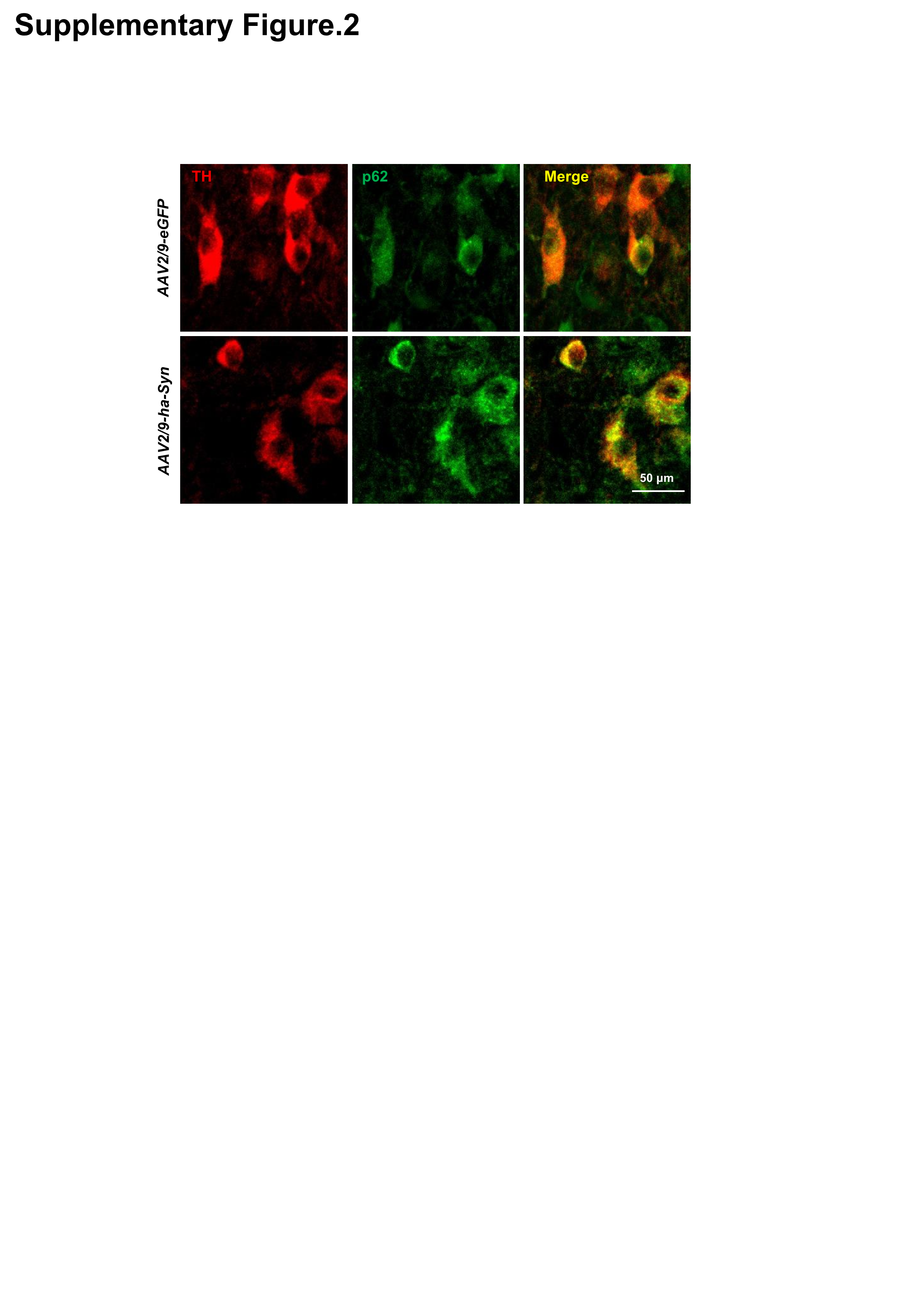

Supplement: Supplementary file 2 — Fig S2 [file ACEL-20-e13522-s005.Tiff]

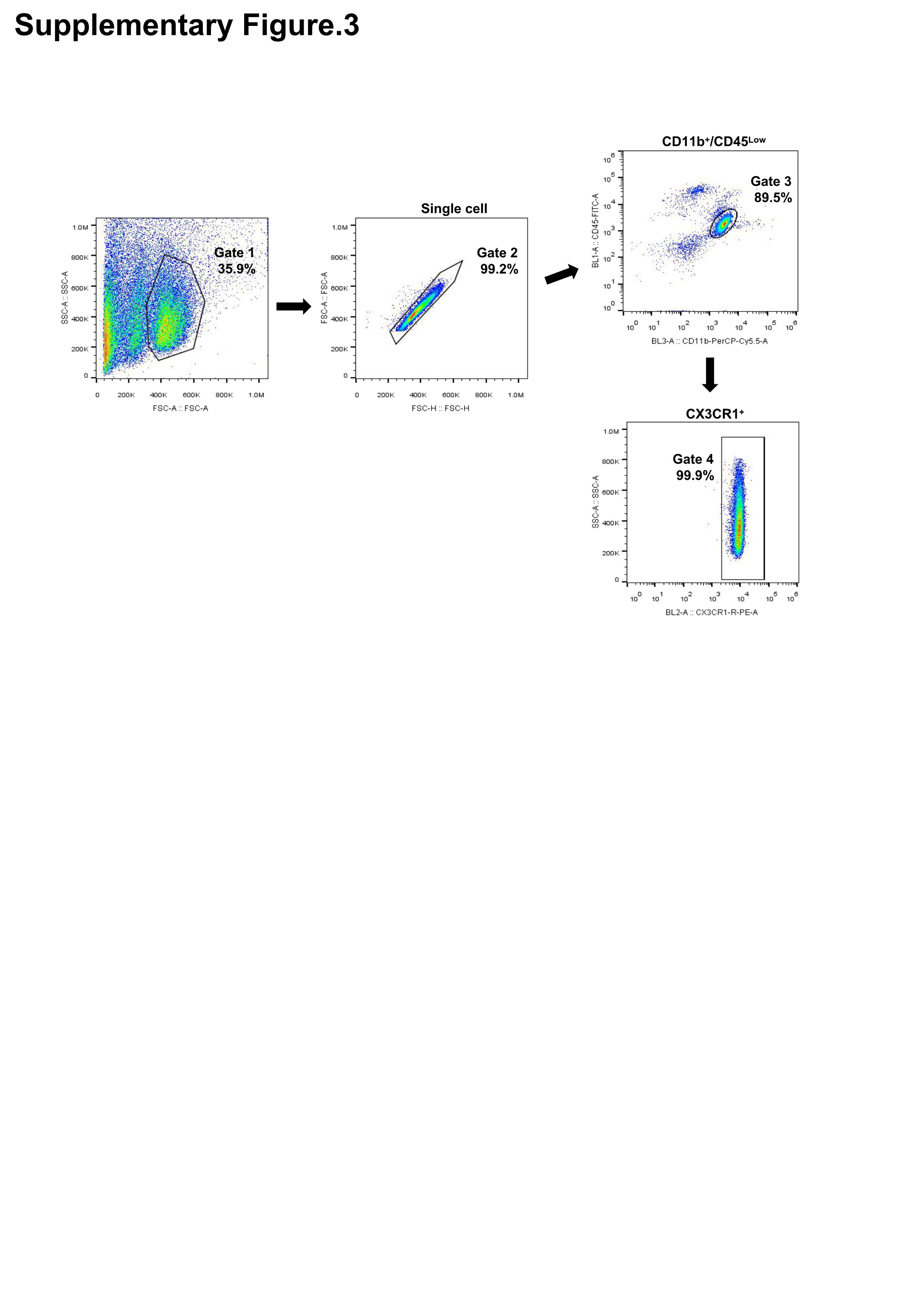

Supplement: Supplementary file 3 — Fig S3 [file ACEL-20-e13522-s004.Tiff]

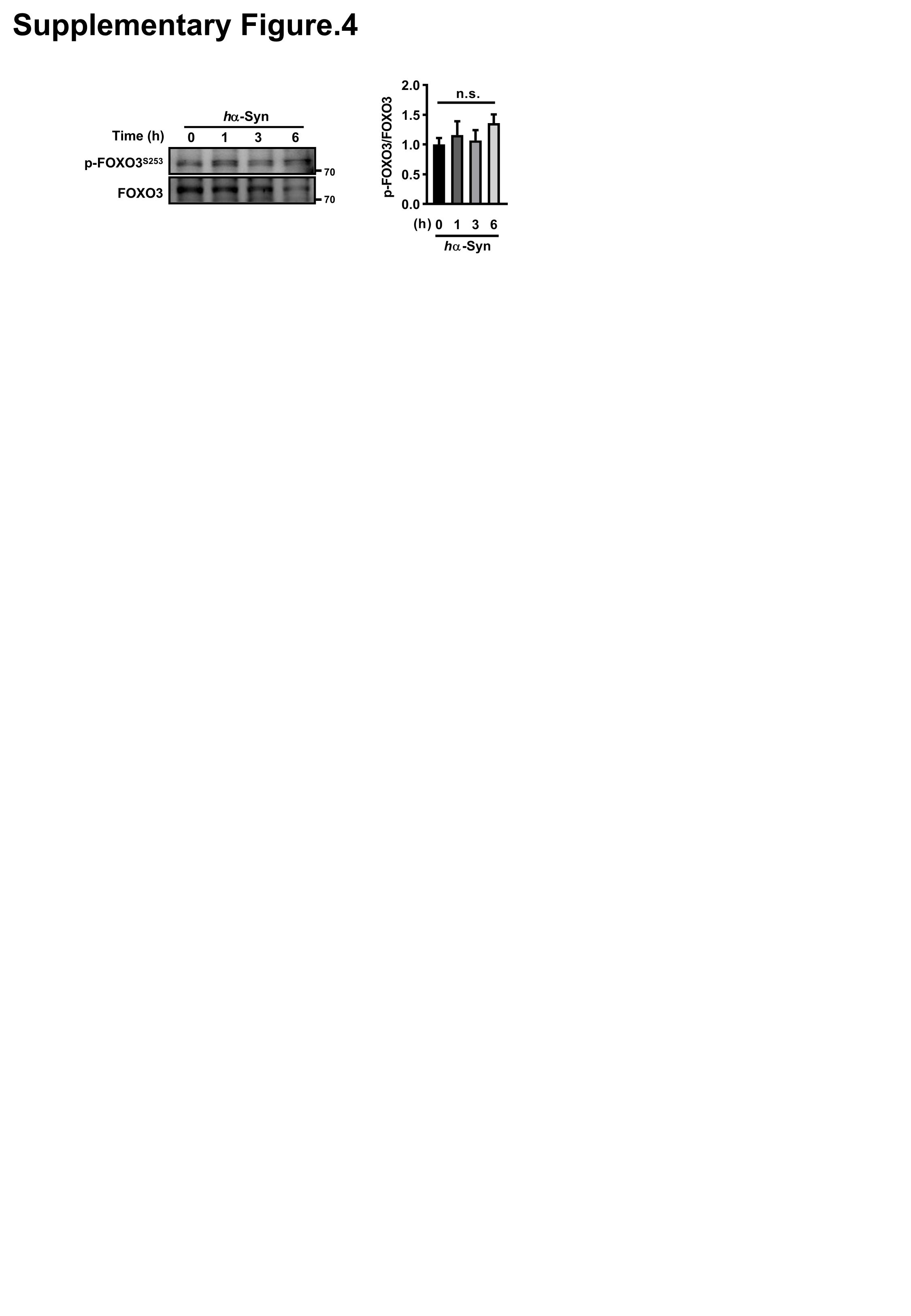

Supplement: Supplementary file 4 — Fig S4 [file ACEL-20-e13522-s006.Tiff]

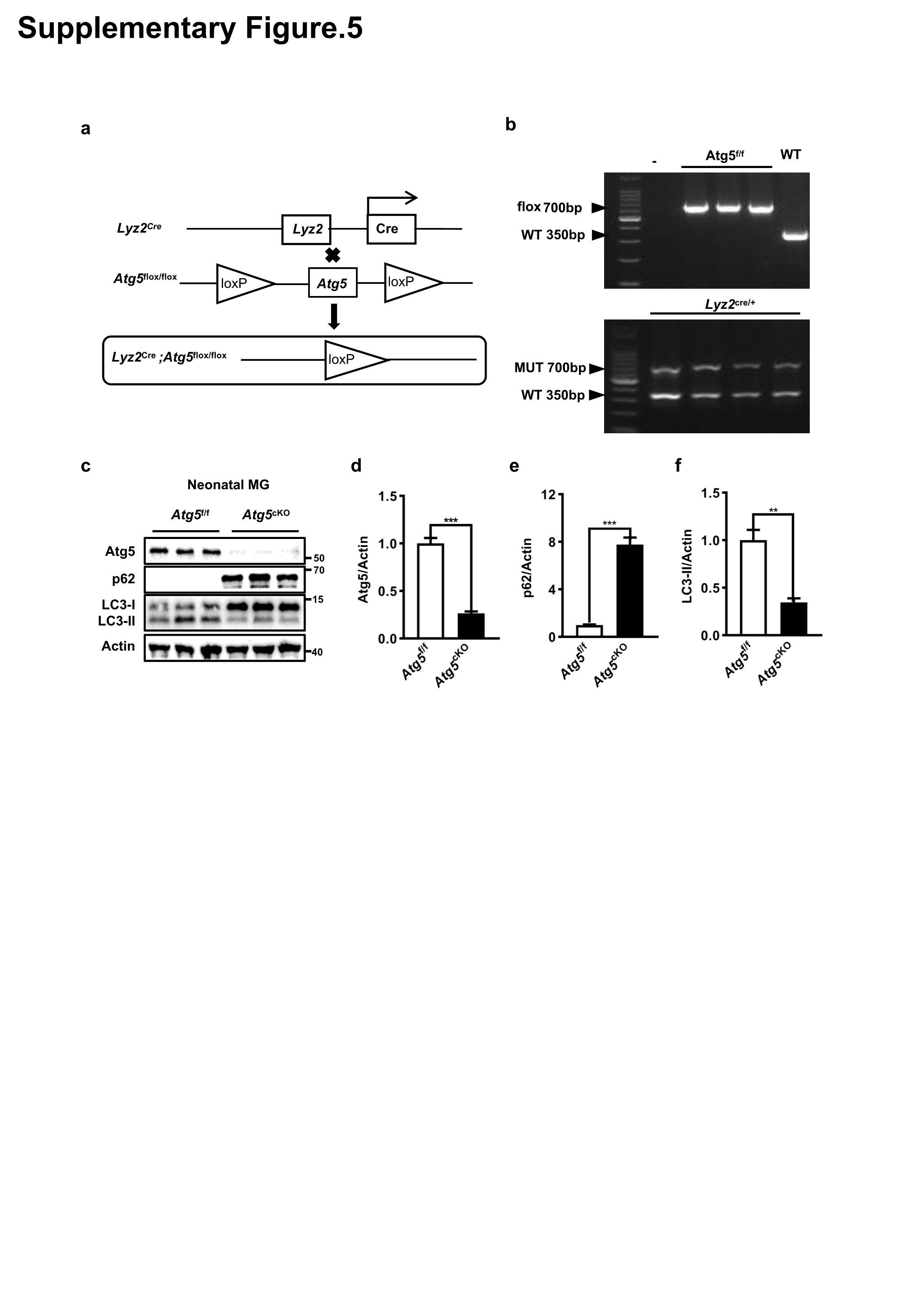

Supplement: Supplementary file 5 — Fig S5 [file ACEL-20-e13522-s008.Tiff]

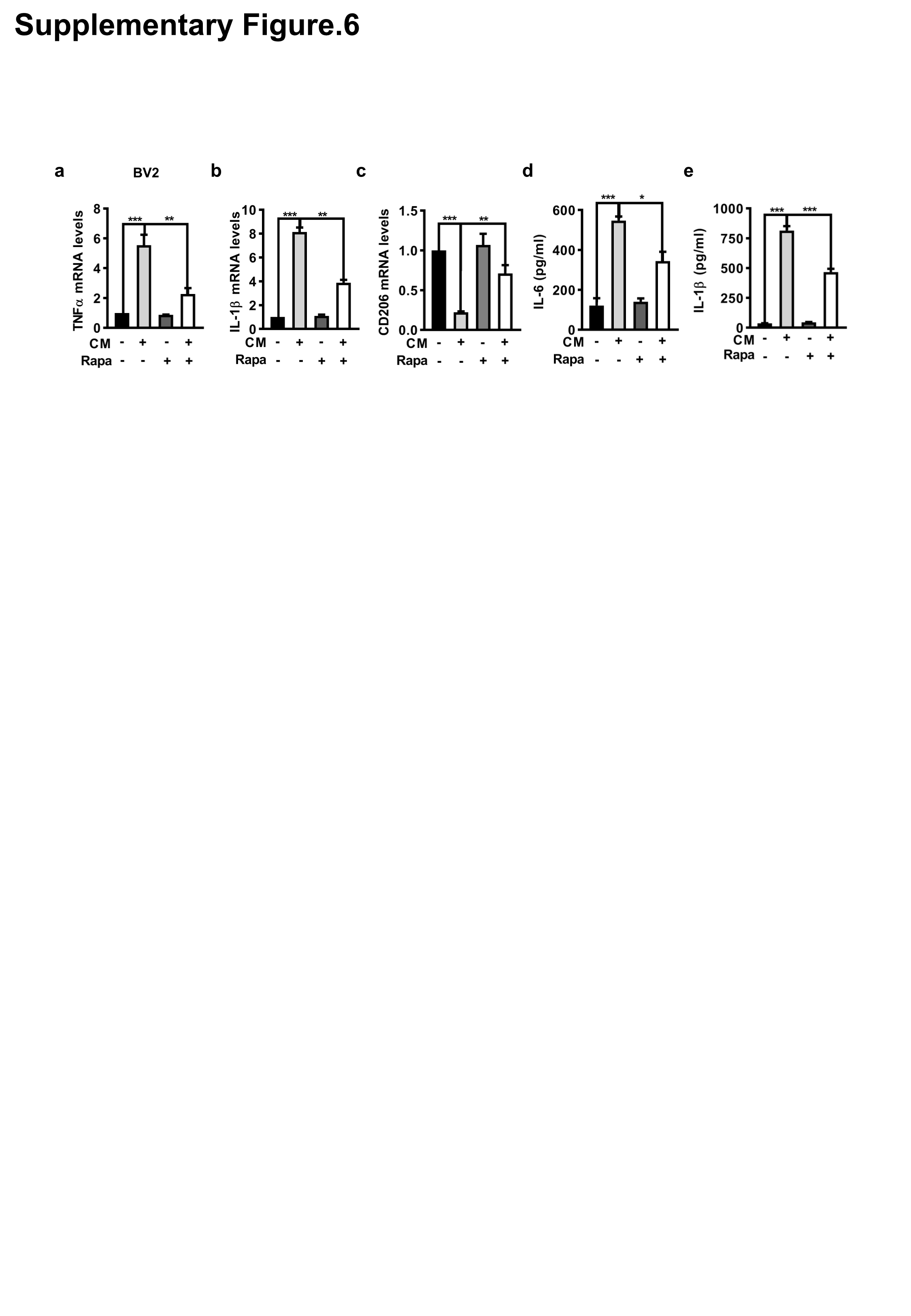

Supplement: Supplementary file 6 — Fig S6 [file ACEL-20-e13522-s002.Tiff]

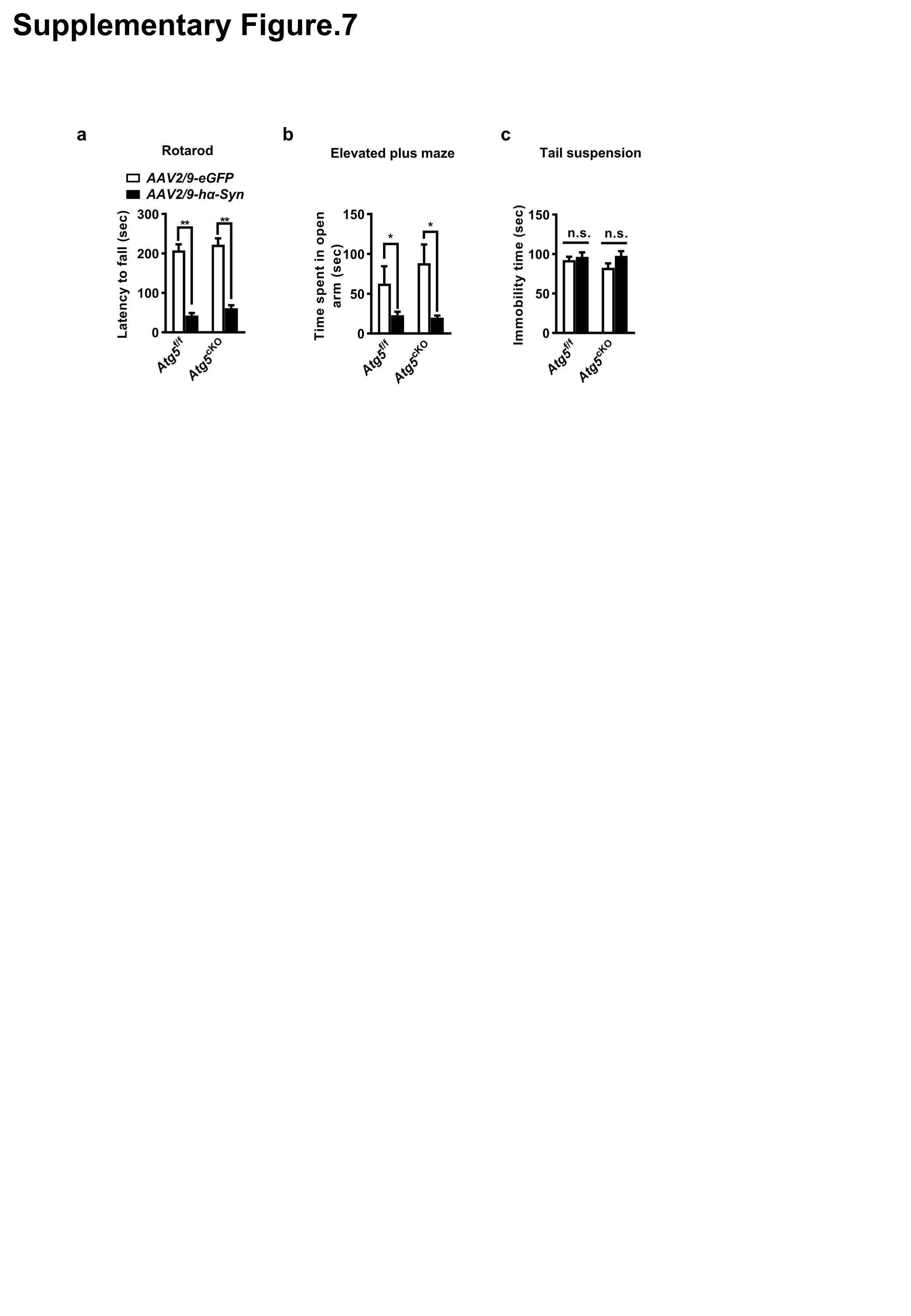

Supplement: Supplementary file 7 — Fig S7 [file ACEL-20-e13522-s001.Tiff]
