# Supplementary material for: α‐synuclein suppresses microglial autophagy and promotes neurodegeneration in a mouse model of Parkinson’s disease
Source: Aging Cell. 2021 Nov 22;20(12):e13522. doi: 10.1111/acel.13522 (PMC8672776; doi:10.1111/acel.13522)
Supplement: Supplementary file 8 — Supplementary Material [file ACEL-20-e13522-s003.doc]

**Supplementary figure legends**

**Figure S1. Characterization of PD mouse model overexpressing human α-Syn.**

**(a)** Bilateral injection with *AAV2/9-hα-Syn or -eGFP* into the SNpc (coordinates relative to bregma: AP: 3.0 mm; ML: 1.25 mm; DV: 4.5 mm.

**(b)** To verify AAV2/9-infected cell type, brain slices of *AAV2/9-eGFP-*injected mice were stained with anti-NeuN, anti-GFAP or anti-Iba1. eGFP fluorescence predominantly localized to NeuN^+^ cells, not GFAP or Iba1 positive cells. In *AAV2/9-hα-Syn*-injected mice, anti-hα-Syn (LB509) positive signal also co-localized to NeuN or TH positive neurons. Scale bar at 100 μm.

**(c)** SN *hα-Syn* expression assessed by western blot using anti-hα-Syn (MJFR1 clone) at 2-, 4- and 8-weeks post injection with *AAV2/9-hα-Syn or -eGFP*.

**(d&e)** DA neuron losses in the SNpc at 8 weeks post injection, indicated by TH^+^ neuron number changes. Scale bar, 200 μm. N=7 brains per group, Student *t* test, ****P* < 0.001.

**Figure S2. Colocalization of p62 with TH positive neurons in the SN.**

Double stained midbrain sections from *AAV2/9-hα-Syn* or *-eGFP* injected mice, using the antibodies against TH and p62. Scale bar, 50 μm.

**Figure S3.** **Validation of the microglia purity isolated from adult mice.**

Microglia-enriched population was isolated from adult mice brains (3-4 brains pooled for one sample) using the Percoll gradient separation method, and incubated with the surface marker antibodies individually conjugated with different fluorescent tags. The flow cytometry analysis showed that about 89.5% of the single cell population (gate 2) presented CD11b^+^/CD45^low/intermediate^ (gate 3), among which 99% was CX3CR1^+^ cells (gate 4).

**Figure S4. Phosphorylation of FOXO3 in *h*α-Syn treated primary microglia.**

*h*α-Syn treated primary microglia for 1, 3 and 6 h. Western blot analysis for the phosphorylations of p-FOXO3 (S253), normalized to total FOXO3 level, n=4. One-way ANOVA with *Dunnett's post hoc*, n.s., not significant.

**Figure S5.** **Construction of microglia *Atg5*-deficient mice.**

**(a&b)** Strategy for microglia *Atg5*-deficient mice construction. Representative genotyping results shown in (b). WT, wildtype C57BL/6 mice.

**(c-f)** Characterization of *Atg5* deficiency in primary microglia culture prepared from neonatal *Atg5*^f/f^ and *Atg5*^cKO^ mice by western blot study. Student *t* test, n=4, ***P* < 0.01, ****P* < 0.001.

**Figure S6.** **Autophagy modulated the inflammation in microglia.**

**(a-e)** BV2 were treated with *h*α-Syn-CM for 6 h with or without rapamycin (Rapa) (5 μM) co-treatment. The mRNA levels of *TNF-α* (a, n=3), *IL-1β* (b, n=5) and *CD206* (c, n=4) were determined by quantitative PCR analysis. Supernatant IL-6 (d, n=3) and IL-1β (e, n=5) levels were measured by ELISA. One-way ANOVA with *Tukey's post hoc*, **P* < 0.05, ***P* < 0.01, ****P* < 0.001.

**Figure S7. Non-motor deficits in *AAV2/9-hα-Syn* injected microglia *Atg5*-deficient mice.**

**(a)** Rotarod test showing the latency to fall off the rotarod recorded in seconds.

**(b)** The time spent in the open arms in the elevated plus maze.

**(c)** The immobility time during tail suspension test.

*AAV2/9-eGFP*-injected groups (n=10 mice for each genotype) and *AAV2/9-hα-syn-injected* groups (n=8 mice for each genotype). Two-way ANOVA with *Tukey's* *post hoc*, **P* < 0.05, ***P* < 0.01, n.s., not significant.

**Supplementary methods**

**1. Flow cytometry**

Microglia-enriched suspension was incubated with anti-mouse CD16/CD32 (101319, Biolegend) on ice for 30 min to block Fc-receptor, followed by FITC-conjugated CD45 (103107, Biolegend), with PerCP/Cyanine 5.5-conjugated CD11b (101227, Biolegend) and with PE-conjugated CX3CR1 (149005, Biolegend) incubation for 30 min. After brief washes, the microglia population presenting CD11b^+^/CD45^low^/CX3CR1^+^ was gated as shown in Fig. S3. In the microglia-enriched cell suspension, the percentage of CD11b^+^/CD45^low^ population reached about 89.5%, among which 99% was CX3CR1^+^ cells, qualified as adult microglia population for further study.

**2. Elevated plus maze.**

The elevated plus maze consisted of an open arm (60 cm X 5cm) and a closed arm with a wall (60 cm X 5cm) connected to a central zone (5 cm X 5 cm) and was elevated to a height of 50 cm above the floor. Each mouse was placed in the central intersection facing an open arm at the beginning of the task. The video camera was suspended above to record the mice track. The time spent in the open arm was recorded within a 5-min test session.

**3. Tail suspension test.**

The mice were individually suspended by the tail using an adhesive tape placed approximately 1 cm from the tip of the tail attached to a hook and hanging 5 cm above the floor. The immobility time was recorded during a 5-min test session.

**4. Rotarod test.**

The mice were trained once daily three days before test. On day 4, mice were placed on an accelerating rotarod cylinder at the speed from 4 to 40 rpm within 5 min. A trial ended if the mouse fell off the rod or just gripped the device without attempting to run on the rungs. And the latency time to fall off the rod was recorded. Each mouse was subjected to three trials, with a 10-min interval, and the mean value of the latency time during three trials was calculated and presented for each animal.
